# Supplementary material for: Emulation of coherent absorption of Fock-state quantum light in a programmable linear photonic circuit
Source: Nat Commun. 2026 May 9;17:4211. doi: 10.1038/s41467-026-72850-6 (PMC13157496; doi:10.1038/s41467-026-72850-6)
Supplement: Supplementary file 1 — Supplementary Information [file 41467_2026_72850_MOESM1_ESM.pdf]

Supplementary Materials: Emulation of Coherent Absorption of Fock-State Quantum Light in a Programmable Linear Photonic Circuit.

Govind Krishna,[1,*] Jun Gao,[1,2*] Sam O'Brien,[1] Rohan Yadgirkar,[1] Venkatesh Deenadayalan,[3] Stefan Preble,[3] Val Zwiller,[1] & Ali W. Elshaari[1,*]

[1]Department of Applied Physics, KTH Royal Institute of Technology, Albanova University Centre, Roslagstullsbacken 21, 106 91 Stockholm, Sweden
[2]School of Optical and Electronic Information, Huazhong University of Science and Technology, Luoyu Road 1037, Wuhan, Hubei, 430074, China
[3]Microsystems Engineering, Rochester Institute of Technology, Rochester, New York 14623, USA

[*]E-mail: govindk@kth.se, jungao@hust.edu.cn, elshaari@kth.se

Supplementary Note 1 - Construction of the port symmetric lossy beamsplitter transformation matrix

1.1 - Determination of t and r from a target absorption coefficient $|A|^2$

We consider a port-symmetric 2×2 lossy beamsplitter with scattering matrix

$$S = \begin{pmatrix} t & r \\ r & t \end{pmatrix}, \tag{1}$$

where t and r are complex transmission and reflection coefficients, respectively. The intrinsic absorption coefficient $\alpha := |A|^2 \in [0, 0.5]$ satisfies

$$|t|^2 + |r|^2 + \alpha = 1, \qquad 2|t||r| \cos \phi_{rt} = \pm\alpha, \tag{2}$$

with $\phi_{rt} = \arg(r) - \arg(t)$ denoting the internal phase difference. We extract t and r from a given α as follows.

Type 1: Fixed $\phi_{rt} = \pi$.

1. Set $\cos\phi_{rt} = -1$ in Eq. (2).

2. Let $x = |t|^2$, so that $|r|^2 = 1 - \alpha - x$.

3. From Eq. (2), obtain

$$2\sqrt{x(1-\alpha-x)} = \pm\alpha \quad \Rightarrow \quad 4x(1-\alpha-x) = \alpha^2.$$

4. Solve the quadratic and select the branch

$$x = \frac{(1-\alpha) + \sqrt{1-2\alpha}}{2},$$

ensuring $|t| \to 1$ as $\alpha \to 0$. If the other branch is selected, we get the solutions satisfying $|r| \to 1$ as $\alpha \to 0$

5. Assign

$$|t| = \sqrt{x}, \quad |r| = \sqrt{1-\alpha-x}, \quad t = |t|, \quad r = -|r| = |r|e^{i\pi}.$$

Type 2: Equal magnitudes $|t| = |r|$.

1. Impose $|t| = |r| = \sqrt{x}$

2. The first constraint gives

$$2x = 1 - \alpha \quad \Rightarrow \quad x = \frac{1-\alpha}{2}.$$

3. From the second constraint,

$$\cos\phi_{rt} = \frac{\pm\alpha}{2x} = \pm\frac{\alpha}{1-\alpha}.$$

4. We consider the term with the negative sign and extract ϕ_{rt}

$$\phi_{rt} = \arccos\left(-\frac{\alpha}{1-\alpha}\right), \quad \phi_{rt} \in [0, \pi]$$

5. Assign

$$t = \sqrt{x}, \quad r = \sqrt{x}\, e^{i\phi_{rt}}.$$

Remarks. For Type 1, the magnitudes of t and r vary with α, recovering a fully transparent device ($|t| \to 1$, $|r| \to 0$) as $\alpha \to 0$. For Type 2, the magnitudes are fixed at $|t| = |r|$, and it is the relative phase ϕ_{rt} that changes with α, tending to $\pi/2$ as $\alpha \to 0$.

1.2 - Rank of the loss matrix and minimal ancilla dimension

For a passive linear optical transformation T, the evolution can be embedded into a larger unitary operation by introducing a loss coupling matrix L that describes coupling to unobserved modes. Preservation of the bosonic commutation relations then requires

$$T^{\dagger}T + L^{\dagger}L = I, \tag{3}$$

so that the loss contribution is fully characterized by the positive semidefinite matrix

$$M \equiv I - T^{\dagger}T. \tag{4}$$

For the reciprocal, port-symmetric beam splitter considered here, $T = \begin{pmatrix} t & r \\ r & t \end{pmatrix}$, the constraints derived above,

$$|t|^2 + |r|^2 + |A|^2 = 1, \qquad 2|t||r|\cos\phi_{rt} = |A|^2,$$

or equivalently $tr^* + rt^* = |A|^2$, yield

$$M = |A|^2 \begin{pmatrix} 1 & 1 \\ 1 & 1 \end{pmatrix}. \tag{5}$$

This matrix has a single nonzero eigenvalue and therefore

$$\mathrm{rank}(I - T^{\dagger}T) = 1. \tag{6}$$

Since the eigenvalues of $M = I - T^{\dagger}T$ are given by $1 - \sigma_i^2$, where σ_i denote the singular values of T, the rank of M equals the number of singular values of T that differ from unity. For

a passive transformation, all singular values satisfy $\sigma_i \leq 1$, so that exactly one singular value of T is strictly smaller than unity in the present case. Within the quasi-unitary dilation framework, the minimal number of ancilla modes required to embed a passive non-unitary transformation equals the number of singular values of T that are strictly smaller than 1, i.e.

$$m_{\text{anc}} = \text{rank}(I - T^{\dagger}T). \tag{7}$$

Thus, a single ancilla mode is both necessary and sufficient for the port-symmetric CPA transformations implemented in this work.

For more general lossy beam splitters, this rank-1 condition does not necessarily hold. If port symmetry is relaxed-for example, when the two ports couple to distinct loss channels or when absorption is asymmetric, the matrix $I - T^{\dagger}T$ is generically full rank. In such cases, both singular values of a 2×2 passive non-unitary transformation are strictly smaller than unity, yielding

$$\text{rank}(I - T^{\dagger}T) = 2,$$

and the minimal unitary dilation therefore requires two ancilla modes. More generally, for any passive linear optical transformation, the number of ancilla modes required in a quasi-unitary embedding is determined by the number of singular values of T that differ from unity, i.e. $m_{\text{anc}} = \text{rank}(I - T^{\dagger}T)$.

For an extended lossy network composed of multiple lossy 2×2 units, one may first construct the overall passive non-unitary transformation matrix T_{net} of the full device by composing the individual blocks on the appropriate mode subspaces. The minimal ancilla dimension is then fixed by $m_{\text{anc}} = \text{rank}(I - T_{\text{net}}^{\dagger}T_{\text{net}})$, that is, by the rank of the *net* loss matrix rather than by the number of lossy elements used in the internal decomposition. While a strictly planar geometry may impose practical routing constraints if one attempts to associate independent ancilla modes with each individual lossy beam-splitter unit in a large mesh, this does not limit the general-

ity of the approach, since the same physical operation can be realized by embedding the net transformation T_{net} directly using the ancilla-assisted procedure demonstrated in this work.

Supplementary Note 2 - Derivation of MZI Phase Conditions

NB: Throughout this section, we use the notations θ_i and ϕ_i interchangeably with $\theta_{\mathrm{MZI}i}$ and ϕ_{MZI_i} for brevity and to reduce typographical clutter.

2.1 - Derivation of θ_{MZI_2} from Absorptivity

MZI_2 in the CPA interferometer sets the effective absorption coefficient $|A|^2$ of the simulated lossy beamsplitter by routing part of the input light into the ancilla mode. As indicated in the main manuscript Figure 2(b), MZI_2 receives input from output moode 2 of MZI_1 and splits it between the signal and ancilla outputs via an SU(2) transformation. Its action on the input can be expressed as:

$$\begin{pmatrix} \alpha_{\mathrm{S}} \\ \alpha_{\mathrm{anc}} \end{pmatrix} = e^{i\left(\frac{\theta_2}{2}+\frac{\pi}{2}\right)} \begin{pmatrix} e^{i\phi_2}\sin\left(\frac{\theta_2}{2}\right) & \cos\left(\frac{\theta_2}{2}\right) \\ e^{i\phi_2}\cos\left(\frac{\theta_2}{2}\right) & -\sin\left(\frac{\theta_2}{2}\right) \end{pmatrix} \begin{pmatrix} \alpha \\ 0 \end{pmatrix}, \tag{8}$$

where α is the complex amplitude of the field entering MZI_2 from MZI_1, and θ_2 and ϕ_2 are the internal and external phase shifts of MZI_2, respectively.

The complex amplitude at the ancilla port is then:

$$\alpha_{\mathrm{anc}} = e^{i\left(\frac{\theta_2}{2}+\frac{\pi}{2}\right)} e^{i\phi_2} \cos\left(\frac{\theta_2}{2}\right) \alpha. \tag{9}$$

Taking the intensity at the ancilla output:

$$|\alpha_{\mathrm{anc}}|^2 = |\alpha|^2 \cos^2\left(\frac{\theta_2}{2}\right), \tag{10}$$

We now aim to write the expression for the ancilla intensity entirely in terms of the intrinsic absorption coefficient $|A|^2$, using the beam splitter scattering relations and the constraint equations. The non-unitary transformation of coherent absorption can be expressed in matrix form as:

$$\begin{bmatrix} t & r \\ r & t \end{bmatrix} \times \frac{1}{\sqrt{2}} \begin{bmatrix} e^{i\phi} \\ -1 \end{bmatrix} = \frac{1}{\sqrt{2}} \begin{bmatrix} te^{i\phi} - r \\ re^{i\phi} - t \end{bmatrix},$$

where ϕ is the phase of the input quantum state

Absorbed intensity:

$$\begin{aligned}
I_{\text{abs}} &= 1 - \frac{1}{2}\left|te^{i\phi} - r\right|^2 - \frac{1}{2}\left|re^{i\phi} - t\right|^2 \\
&= 1 - \frac{1}{2}\left[(te^{i\phi} - r)(t^*e^{-i\phi} - r^*) + (re^{i\phi} - t)(r^*e^{-i\phi} - t^*)\right] \\
&= 1 - \frac{1}{2}\left[|t|^2 - rt^*e^{-i\phi} - r^*te^{i\phi} + |r|^2 + |r|^2 - tr^*e^{-i\phi} - t^*re^{i\phi} + |t|^2\right] \\
&= 1 - |t|^2 - |r|^2 - \frac{1}{2}\left[e^{i\phi} + e^{-i\phi}\right]\left[tr^* + rt^*\right] \\
&= |A|^2 - \cos\phi\left[tr^* + rt^*\right] \\
&= |A|^2 - \cos\phi\left[2|t||r|cos\phi_{rt}\right] \\
&= |A|^2 \pm |A|^2\cos\phi.
\end{aligned} \tag{11}$$

Thus, at the input state phases ϕ corresponding to maximal absorption, the intensity in the ancilla mode, i.e, the absorbed intensity, is given by $2|A|^2$. Recall that full light absorption is achieved when $|A|^2 = 0.5$ (see the theoretical and experimental curves in 3(b) and 3(e) of the main text), which is also the fundamental upper bound for the intrinsic absorption coefficient of a port-symmetric lossy beamsplitter (see Methods). At these ϕ values, the light emerging from MZI_1 is directed entirely into the input mode 1 of MZI_2. We therefore set Eq. 10 equal to $2|A|^2$ at $\alpha = 1$ and obtain:

$$2|A|^2 = \cos^2\left(\frac{\theta_2}{2}\right). \tag{12}$$

Solving for θ_2, we arrive at the expression:

$$\boxed{\theta_{\mathrm{MZI}_2} = \theta_2 = 2\cos^{-1}\left(\sqrt{2|A|^2}\right),} \tag{13}$$

as used in the main text. This provides a direct mapping between the desired $|A|^2$ and the internal phase setting of MZI_2. As an experimental validation of Eq. 13, we measure the maximum ancilla-mode probability at the input phase corresponding to perfect absorption and observe the predicted relation $P_{\mathrm{ancilla}} = 2|A|^2$, shown in Supplementary Figure 1.

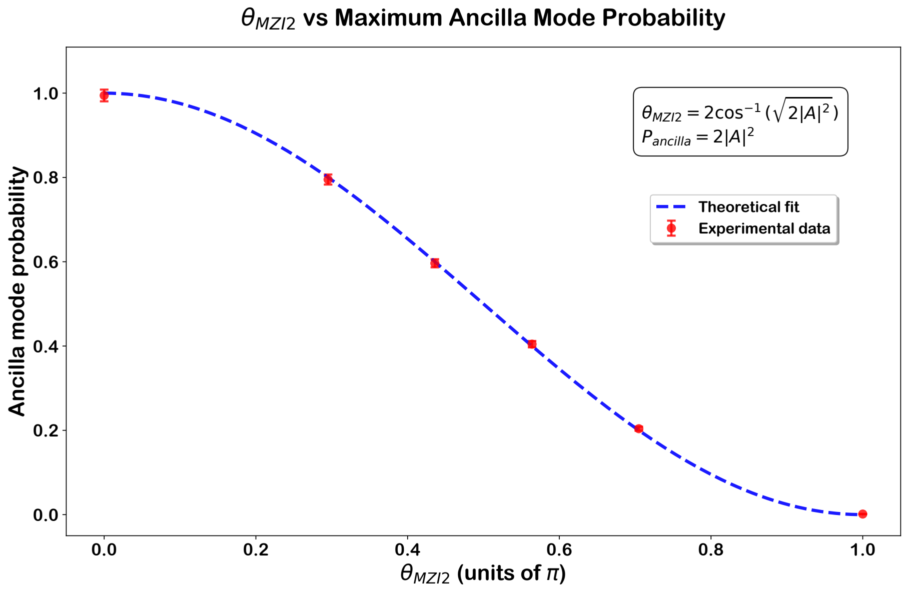

Supplementary Figure 1: **Experimental validation of Eq. 13.** With the input phase set to the perfect absorption condition, the measured maximum ancilla-mode probability follows $P_{\mathrm{ancilla}} = 2|A|^2$, yielding $\theta_{\mathrm{MZI2}} = 2\cos^{-1}(\sqrt{2|A|^2})$. Markers: experimental data. Line: theoretical prediction.

2.2 - Derivation of the Phase Condition for $\phi_{\mathrm{MZI}_3} - \phi_{\mathrm{MZI}_2}$

To derive the relative phase condition between the external phase shifters of MZI_2 and MZI_3, we analytically evaluate the 3-mode unitary transformation implemented by the CPA interferometer

circuit. For the ease of calculation, we consider only the transformation of the first two modes (signal1 and signal2). This derivation uses the form of the MZI unitary already introduced in the main manuscript (Eq.13), repeated here for convenience:

$$U_{\mathrm{MZI}}(\theta, \phi) = e^{i\left(\frac{\theta}{2}+\frac{\pi}{2}\right)} \begin{pmatrix} e^{i\phi}\sin\left(\frac{\theta}{2}\right) & \cos\left(\frac{\theta}{2}\right) \\ e^{i\phi}\cos\left(\frac{\theta}{2}\right) & -\sin\left(\frac{\theta}{2}\right) \end{pmatrix}. \tag{14}$$

As mentioned in the main text, throughout all beam splitter circuit configurations, the following phase shift values are constant

$$\theta_1 = \theta_3 = \frac{\pi}{2}, \quad \phi_1 = -\pi$$

Also, we have already shown in the previous section that

$$\theta_2 = 2\cos^{-1}\left(\sqrt{2|A|^2}\right)$$

We start by evaluating the Scattering matrices step by step.

MZI1:

$$U(\mathrm{MZI}_1) = \frac{C}{\sqrt{2}} \begin{pmatrix} -1 & 1 \\ -1 & -1 \end{pmatrix}. \tag{15}$$

where we define: $C = \exp\left(i\frac{3\pi}{4}\right)$.

MZI2: Acts only on modes 2 and 3. Here, we define a scattering matrix that describes its action on modes 1 and 2, as these are the modes of interest. The scattering matrix defining this transformation is non-unitary (as some input light is lost to mode 3 at this step and we don't take it into account in this matrix) and is of the form:

$$S(\mathrm{MZI}_2) = \begin{pmatrix} 1 & 0 \\ 0 & B \end{pmatrix}, \tag{16}$$

where:

$$B = \exp\left[i\left(\frac{\theta_2}{2} + \frac{\pi}{2}\right)\right] e^{i\phi_2} \cdot \sqrt{1 - 2|A|^2}. \tag{17}$$

MZI$_3$:

$$U(\mathrm{MZI}_3) = C \cdot \begin{pmatrix} \frac{e^{i\phi_3}}{\sqrt{2}} & \frac{1}{\sqrt{2}} \\ \frac{e^{i\phi_3}}{\sqrt{2}} & -\frac{1}{\sqrt{2}} \end{pmatrix}, \tag{18}$$

Now we perform the matrix multiplications:

Step 1: $S_{21} = S(\mathrm{MZI}_2) \cdot U(\mathrm{MZI}_1)$

$$S_{21} = \begin{pmatrix} 1 & 0 \\ 0 & B \end{pmatrix} \cdot \frac{C}{\sqrt{2}} \begin{pmatrix} -1 & 1 \\ -1 & -1 \end{pmatrix} = \frac{C}{\sqrt{2}} \begin{pmatrix} -1 & 1 \\ -B & -B \end{pmatrix}. \tag{19}$$

Step 2: $S_{\textbf{total}} = U(\mathrm{MZI}_3) \cdot S_{21}$

$$S_{\text{total}} = C \cdot \begin{pmatrix} \frac{e^{i\phi_3}}{\sqrt{2}} & \frac{1}{\sqrt{2}} \\ \frac{e^{i\phi_3}}{\sqrt{2}} & -\frac{1}{\sqrt{2}} \end{pmatrix} \cdot \frac{C}{\sqrt{2}} \begin{pmatrix} -1 & 1 \\ -B & -B \end{pmatrix} \tag{20}$$

$$= \frac{C^2}{2} \begin{pmatrix} -e^{i\phi_3} - B & e^{i\phi_3} - B \\ -e^{i\phi_3} + B & e^{i\phi_3} + B \end{pmatrix}. \tag{21}$$

The Clements decomposition process used to implement this interferometer circuit introduces additional output phases at the end of all output modes of the circuit (see Supplementary Figure 2). These are mode-dependent but consistent across instances of a given decomposition. We denote them by δ_1 and δ_2, which are added to the output of modes 1 and 2, respectively. In our simulations, $\delta_2 - \delta_1 = \pm\pi$ always holds, though the absolute values vary with each decomposition. This was computationally verified by independently performing quasi-unitary extension and Clements decomposition on 10,000 randomly generated port-symmetric lossy beamsplitter matrices.

These phases are modeled as an additional diagonal unitary:

$$D = \mathrm{diag}(e^{i\delta_1},\ e^{i\delta_2}) = \mathrm{diag}(e^{i\delta_1},\ e^{i\delta_1 \pm \pi}) = \mathrm{diag}(e^{i\delta_1},\ -e^{i\delta_1}). \tag{22}$$

Thus, the final transformation matrix becomes:

$$S_{\text{eff}} = D \cdot S_{\text{total}} = \begin{pmatrix} e^{i\delta_1} & 0 \\ 0 & -e^{i\delta_1} \end{pmatrix} \cdot S_{\text{total}}. \tag{23}$$

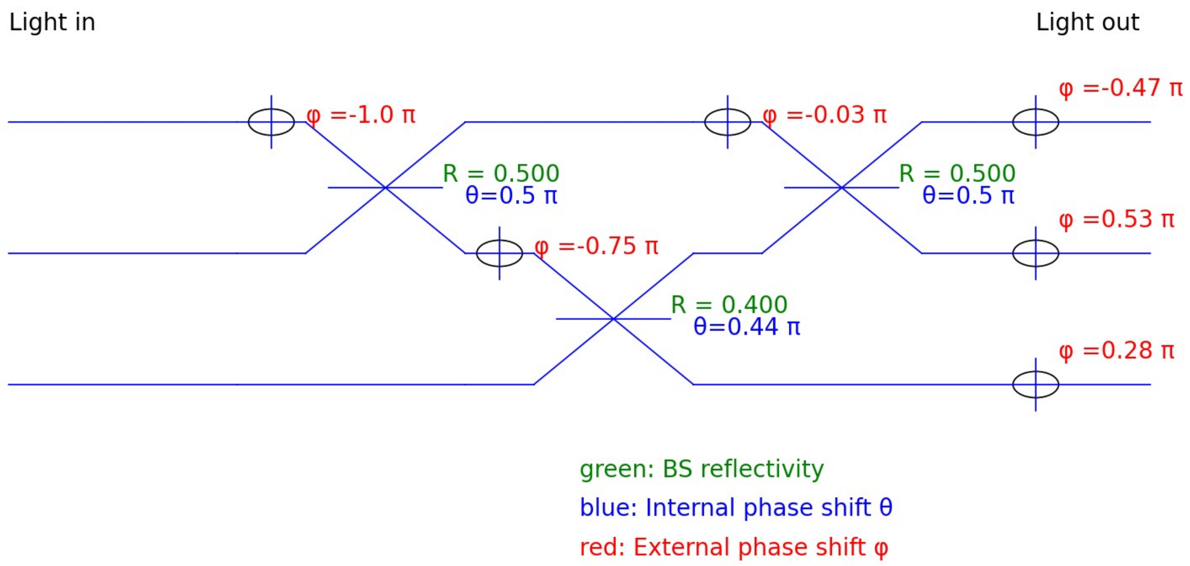

Supplementary Figure 2: **Clements decomposition for the CPA circuit** corresponding to $|A|^2 = 0.3$ and $\phi_{rt} = \pi$. The decomposition provides internal (θ) and external (ϕ) phase shift values for each MZI in the mesh, which are used to implement the desired 3×3 unitary. The additional output phase shifts visible at the end of each mode (e.g., $\phi = 0.53\pi$ and $\phi = -0.47\pi$) arise from the decomposition and are absorbed into the diagonal matrix D in the theoretical model.

$$S_{\text{eff}} = \frac{C^2}{2} \begin{pmatrix} -e^{i\delta_1}(e^{i\phi_3} + B) & e^{i\delta_1}(e^{i\phi_3} - B) \\ e^{i\delta_1}(e^{i\phi_3} - B) & -e^{i\delta_1}(e^{i\phi_3} + B) \end{pmatrix}. \tag{24}$$

Now, we match this matrix, which represents the total circuit transformation to the target lossy beamsplitter matrix:

$$S = \begin{pmatrix} t & r \\ r & t \end{pmatrix}, \tag{25}$$

we identify the first row elements as:

$$t = -\frac{C^2}{2} e^{i\delta_1}(e^{i\phi_3} + B), \tag{26}$$

$$r = \frac{C^2}{2} e^{i\delta_1}(e^{i\phi_3} - B). \tag{27}$$

Adding and subtracting these gives:

$$t + r = -C^2 e^{i\delta_1} B, \tag{28}$$

$$t - r = -C e^{i\delta_1} e^{i\phi_3}. \tag{29}$$

Hence,

$$\frac{t+r}{t-r} = \frac{B}{e^{i\phi_3}}. \tag{30}$$

Using the definition of B, we substitute:

$$\frac{t+r}{t-r} = \exp[i(\phi_2 - \phi_3)] \cdot \exp\left(i\left(\frac{\theta_2}{2} + \frac{\pi}{2}\right)\right)\sqrt{1 - 2|A|^2}. \tag{31}$$

Taking the argument, we obtain

$$\boxed{\phi_{\mathrm{MZI}_3} - \phi_{\mathrm{MZI}_2} = \phi_3 - \phi_2 = -\arg\left(\frac{t+r}{t-r}\right) + \frac{\theta_{\mathrm{MZI}_2}}{2} + \frac{\pi}{2}} \tag{32}$$

We now aim to express the phase difference $\phi_3 - \phi_2$ purely in terms of the reflection and transmission amplitudes $|t|$ and $|r|$ and their relative phase ϕ_{rt}. Writing $t = |t|e^{i\phi_t}$ and $r = |r|e^{i\phi_r}$, the relative internal phase of the lossy beamsplitter is $\phi_{rt} = \phi_r - \phi_t$. This gives

$$\frac{t+r}{t-r} = \frac{|t|e^{i\phi_t} + |r|e^{i\phi_r}}{|t|e^{i\phi_t} - |r|e^{i\phi_r}} = \frac{|t| + |r|e^{i\phi_{rt}}}{|t| - |r|e^{i\phi_{rt}}}. \tag{33}$$

The argument of this complex ratio can be expressed in trigonometric form as

$$\arg\left(\frac{a + be^{i\phi}}{a - be^{i\phi}}\right) = -\operatorname{sgn}(\cos\phi)\,\tan^{-1}\left(\frac{2ab\sin\phi}{a^2 - b^2}\right), \tag{34}$$

where the sign function is defined as

$$\operatorname{sgn}(x) = \begin{cases} +1, & x > 0, \\ -1, & x < 0. \end{cases} \tag{35}$$

This form accounts for the fact that, under the constraint $2ab\cos\phi = \pm|A|^2$, the complex ratio occupies different quadrants depending on the sign of $\cos\phi$.

Applying Eq. (34) with $a = |t|$, $b = |r|$, and $\phi = \phi_{rt}$, we obtain

$$\arg\left(\frac{t+r}{t-r}\right) = -\operatorname{sgn}(\cos\phi_{rt})\ \tan^{-1}\left(\frac{2|t||r|\sin(\phi_{rt})}{|t|^2 - |r|^2}\right). \tag{36}$$

Substituting this into the phase condition yields the final expression

$$\boxed{\phi_{\mathrm{MZI}_3} - \phi_{\mathrm{MZI}_2} = \frac{\theta_{\mathrm{MZI}_2}}{2} + \frac{\pi}{2} + \operatorname{sgn}(\cos\phi_{rt})\ \tan^{-1}\left(\frac{2|t||r|\sin(\phi_{rt})}{|t|^2 - |r|^2}\right)} \tag{37}$$

Note: The expression in Eq. (37) is invariant under the transformation $\phi_{rt} \to \phi_{rt} + \pi$, and therefore has an intrinsic periodicity of π in the relative phase ϕ_{rt}.

We further numerically verified using several randomly generated lossy beamsplitter matrices that the analytical phase-setting formulas in Eqs. 13 and 37 reproduce exactly the phase values obtained from the quasi-unitary extension and Clements decomposition used to program the chip (100% agreement).

Supplementary Note 3 - Additional Experimental Setup Details

To provide further clarity on device implementation and control electronics, we include the following elaborations:

3.1 - Fabrication and Layout

Supplementary Figure 3 provides structural and visual details of the fabricated chip, including the layer stack, SEM images of the core photonic elements, and an optical micrograph of the complete 8×8 interferometer mesh.

3.2 - Thermo-optic Phase-Shifter Characterization

The interferometer mesh comprises a total of 56 thermo-optic phase shifters, corresponding to the MZIs in the 8×8 Clements mesh. Each phase shifter was characterized using continuous-

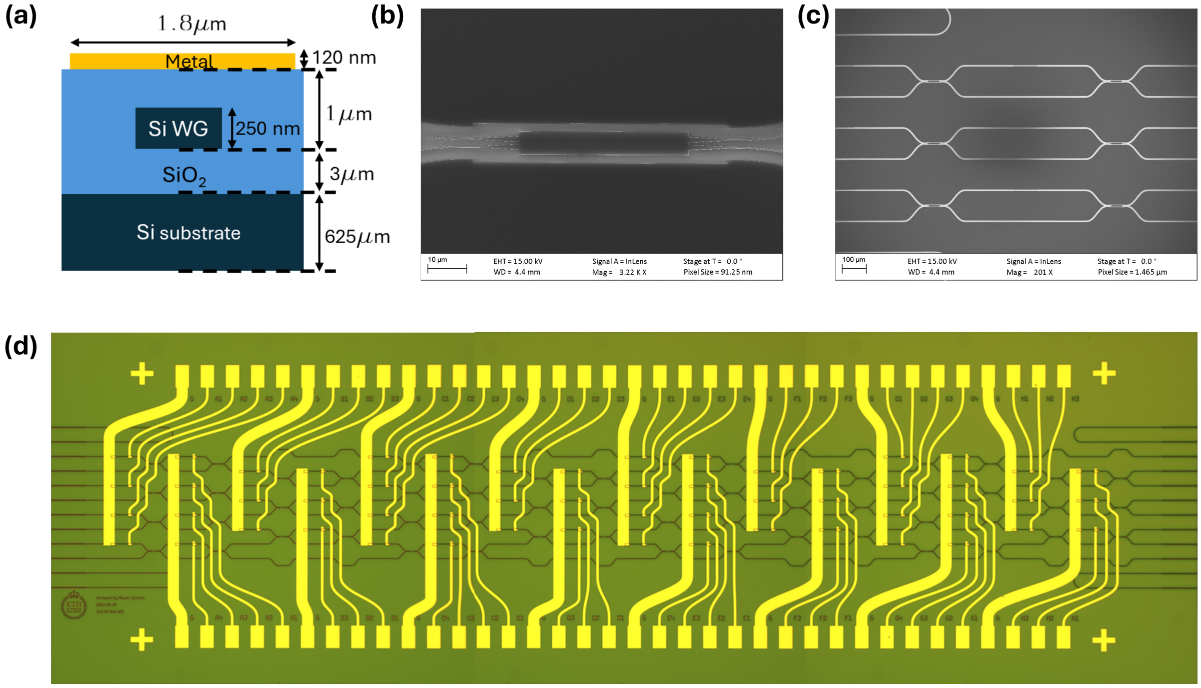

Supplementary Figure 3: **The photonic chip** (a) Schematic cross-section of the photonic chip layer stack. The thermo-optic phase shifters are fabricated using a Ti-based metal heater (120 nm thick, 1.8 μm wide), patterned above a 1 μm SiO_2 cladding layer to minimize absorption. The underlying waveguide core is a 250 nm silicon layer on 3 μm buried oxide atop a 625 μm silicon substrate. (b) SEM image of a single 2×2 multimode interference (MMI) coupler used as a balanced beamsplitter within each MZI. (c) SEM image showing a vertical array of three MZIs forming a single column of the interferometer mesh. (d) Stitched optical micrographs of the entire 8×8 programmable photonic chip, with metal interconnects (yellow) for the thermo-optic phase shifters and edge coupler ports at the chip edge for optical I/O.

wave laser light at 1550 nm and automated Python routines. The internal phase shifters were calibrated using the method described in Ref. [1], while the external phase shifters followed the calibration scheme outlined in Ref. [2].In both calibration schemes, each phase shifter is embedded within an on-chip interferometric loop, where it modulates the optical output power. The output from a selected port is measured as a function of electrical heating power, and the resulting interference fringe is fitted to extract the fringe visibility and π-phase switching power.

The I–V characteristics of the heaters were measured and fitted using a third-order polyno-

mial:

$$V(I) = RI + \beta I^3, \tag{38}$$

where R represents the linear resistance and β accounts for nonlinear thermal effects. The nonlinearity arises from the temperature-dependent resistivity of the heater material, which causes the resistance to vary with increasing current.

The optical modulation response was modeled using a cosine function of the applied electrical power:

$$P(V) = A \cdot \cos(b \cdot P + c) + d, \tag{39}$$

where A is the fringe amplitude, b is the modulation period (i.e., the inverse of the thermal power required to induce a 2π phase shift), c is the phase offset corresponding to zero input power, and d represents the vertical offset, typically equal to half the peak-to-peak modulation amplitude. The fringe visibility was calculated as:

$$\text{Visibility} = \frac{A - d}{d}.$$

Across all characterized phase shifters, we obtained **an average experimental fringe visibility of $\mathbf{0.9984 \pm 0.0003}$ and a mean modulation period of $\mathbf{24.70 \pm 0.03}$ mW**. This corresponds to a mean π-phase switching power of $P_\pi = (24.70 \pm 0.03)\,\mathrm{mW}/2 = 12.35 \pm 0.02\,\mathrm{mW}$. An example I-V trace and optical modulation curve from one phase shifter are shown in Supplementary Figure 4, illustrating high fringe contrast and reliable power-to-phase response.

3.3 - Control Electronics

Thermo-optic phase shifters on the chip are driven using Qontrol Q8-series current driver modules, which provide eight software-defined output channels per module, each capable of delivering up to 24 mA current and 12 V voltage. The drivers support both current and voltage control modes with high precision—current output and sensing are accurate to $\pm 370\,\mathrm{nA}$, and voltage

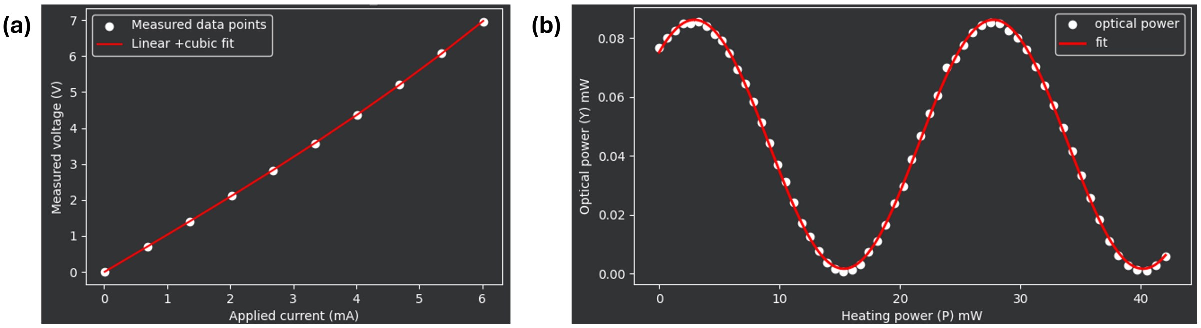

Supplementary Figure 4: **Characterization of one thermo-optic phase shifter.** (**a**) Voltage vs. current from I-V sweep, showing the linear+cubic fit used to extract the non-linear resistance (**b**) Optical output power vs. applied electrical power, fitted with a cosine-squared function.

to $\pm 180\,\mu$V, with 16-bit output and 18-bit input resolution. All channels are individually programmable via USB through a custom Python interface, enabling automated, scalable control of interferometric meshes and active photonic circuits.

More information: https://qontrol.co.uk/product/q8iv/

3.4 - Temperature Stabilization

Temperature control employed a Thorlabs TED200C benchtop TEC controller (range ±2A / 12W) in conjunction with a TH10K thermistor and TECF1S cooler/heater unit. One controller regulated the SPDC crystal temperature, while a second maintained the photonic chip at a constant $25°$C, ensuring stable operation, phase coherence, and wavelength stability.

Supplementary Note 4 - Theoretical formalism of the evolution of the quantum states through the programmable lossy BS interferometric circuit

The evolution of the quantum states through the programmable interferometric circuit can be described using the same 2×2 transformation-matrix formalism we discussed so far. The three Mach–Zehnder interferometers (MZIs) that constitute the circuit each implement a transforma-

tion on the first two modes, represented by the matrices derived in Sec. S2.2 [Eqs. (15)–(18)]. For convenience, these matrices are reproduced below:

$$U(\mathrm{MZI}_1) = \frac{e^{i3\pi/4}}{\sqrt{2}} \begin{pmatrix} -1 & 1 \\ -1 & -1 \end{pmatrix}, \tag{40}$$

$$S(\mathrm{MZI}_2) = \begin{pmatrix} 1 & 0 \\ 0 & e^{i(\frac{\theta_2}{2}+\frac{\pi}{2})} e^{i\phi_2} \sqrt{2|A|^2} \end{pmatrix}, \tag{41}$$

$$U(\mathrm{MZI}_3) = \frac{e^{i3\pi/4}}{\sqrt{2}} \begin{pmatrix} e^{i\phi_3} & 1 \\ e^{i\phi_3} & -1 \end{pmatrix}. \tag{42}$$

4.1 - Single-photon quantum state input.

A single photon entering the circuit is prepared in the dual-rail superposition

$$|\Psi_{1\mathrm{ph}}\rangle = \frac{1}{\sqrt{2}} \big(e^{i\phi} |10\rangle - |01\rangle \big), \tag{43}$$

where $|10\rangle$ and $|01\rangle$ denote one photon in the upper or lower input mode.

Action of MZI$_1$ — The transformation of the field operators across MZI$_1$ defines the orthogonal mode basis for the absorption process. From Eq. (40), the creation operators of the output arms of MZI$_1$, $(\hat{b}_1^\dagger, \hat{b}_2^\dagger)$, are related to the creation operators of the input arms $(\hat{a}_1^\dagger, \hat{a}_2^\dagger)$ by

$$\begin{pmatrix} \hat{b}_1^\dagger \\ \hat{b}_2^\dagger \end{pmatrix} = \frac{e^{-i3\pi/4}}{\sqrt{2}} \begin{pmatrix} -1 & 1 \\ -1 & -1 \end{pmatrix} \begin{pmatrix} \hat{a}_1^\dagger \\ \hat{a}_2^\dagger \end{pmatrix}, \tag{44}$$

so that

$$\hat{b}_1^\dagger = -\frac{e^{-i3\pi/4}}{\sqrt{2}} \big(\hat{a}_1^\dagger - \hat{a}_2^\dagger \big), \qquad \hat{b}_2^\dagger = -\frac{e^{-i3\pi/4}}{\sqrt{2}} \big(\hat{a}_1^\dagger + \hat{a}_2^\dagger \big). \tag{45}$$

Defining the single-photon output arm states $|10\rangle_b = \hat{b}_1^\dagger |\mathrm{vac}\rangle$ and $|01\rangle_b = \hat{b}_2^\dagger |\mathrm{vac}\rangle$, and the input arm states $|10\rangle_a = \hat{a}_1^\dagger |\mathrm{vac}\rangle$, $|01\rangle_a = \hat{a}_2^\dagger |\mathrm{vac}\rangle$, we obtain

$$|10\rangle_b = -\frac{e^{-i3\pi/4}}{\sqrt{2}} \big(|10\rangle_a - |01\rangle_a \big), \qquad |01\rangle_b = -\frac{e^{-i3\pi/4}}{\sqrt{2}} \big(|10\rangle_a + |01\rangle_a \big). \tag{46}$$

Thus, the upper output arm b_1 is a projection of the antisymmetric superposition of the input modes, while the lower output arm b_2 is a projection of the symmetric superposition (up to an overall global phase).

From Eq:(45) we can calculate:

$$\hat{a}_1^\dagger = \frac{e^{i3\pi/4}}{\sqrt{2}}\big(- \hat{b}_1^\dagger - \hat{b}_2^\dagger\big), \qquad \hat{a}_2^\dagger = \frac{e^{i3\pi/4}}{\sqrt{2}}\big(\hat{b}_1^\dagger - \hat{b}_2^\dagger\big). \tag{47}$$

Substituting Eq. (47) into the input state in Eq. (43) gives the state *after* MZI_1 in terms of the b-arms creation operators:

$$\begin{aligned}
\left|\Psi_{1\mathrm{ph}}\right\rangle_{\mathrm{post\ MZI}_1} &= \frac{1}{\sqrt{2}}\Big(e^{i\phi}\hat{a}_1^\dagger - \hat{a}_2^\dagger\Big)\left|\mathrm{vac}\right\rangle \\
&= \frac{1}{\sqrt{2}}\left[e^{i\phi}\,\frac{e^{i3\pi/4}}{\sqrt{2}}\big(- \hat{b}_1^\dagger - \hat{b}_2^\dagger\big) - \frac{e^{i3\pi/4}}{\sqrt{2}}\big(\hat{b}_1^\dagger - \hat{b}_2^\dagger\big)\right]\left|\mathrm{vac}\right\rangle \\
&= \frac{e^{i3\pi/4}}{2}\left[-e^{i\phi}(\hat{b}_1^\dagger + \hat{b}_2^\dagger) - \hat{b}_1^\dagger + \hat{b}_2^\dagger\right]\left|\mathrm{vac}\right\rangle \\
&= \frac{e^{i3\pi/4}}{2}\left[-(e^{i\phi}+1)\hat{b}_1^\dagger + (-e^{i\phi}+1)\hat{b}_2^\dagger\right]\left|\mathrm{vac}\right\rangle \\
&= -\frac{e^{i3\pi/4}}{2}\left[(e^{i\phi}+1)\hat{b}_1^\dagger + (e^{i\phi}-1)\hat{b}_2^\dagger\right]\left|\mathrm{vac}\right\rangle .
\end{aligned} \tag{48}$$

Projection of the input state onto each output arm. — Introducing the arm basis $\left|10\right\rangle_b = \hat{b}_1^\dagger\left|\mathrm{vac}\right\rangle,\ \left|01\right\rangle_b = \hat{b}_2^\dagger\left|\mathrm{vac}\right\rangle$, Eq. (48) can be written as

$$\left|\Psi_{1\mathrm{ph}}\right\rangle_{\mathrm{post\ MZI}_1} = \alpha_1(\phi)\,\left|10\right\rangle_b + \alpha_2(\phi)\,\left|01\right\rangle_b, \tag{49}$$

with the arm amplitudes (projections of the input state onto the upper and lower arms) given by

$$\alpha_1(\phi) = -\frac{e^{i3\pi/4}}{2}\big(e^{i\phi}+1\big), \qquad \alpha_2(\phi) = -\frac{e^{i3\pi/4}}{2}\big(e^{i\phi}-1\big). \tag{50}$$

Post–MZI_1 state in the b-arm basis. — Using $e^{i\phi}+1 = 2e^{i\phi/2}\cos(\phi/2)$ and $e^{i\phi}-1 = 2ie^{i\phi/2}\sin(\phi/2)$, we obtain,

$$\boxed{\left|\Psi_{1\mathrm{ph}}\right\rangle_{\mathrm{post\ MZI}_1} = \big(- e^{i(3\pi/4+\phi/2)}\big)\left[\cos\!\left(\frac{\phi}{2}\right)\left|10\right\rangle_b \,+\, i\,\sin\!\left(\frac{\phi}{2}\right)\left|01\right\rangle_b\right].} \tag{51}$$

Thus, for this particular input state, the upper arm after MZI_1 carries an amplitude proportional to $\cos(\phi/2)$ and the lower arm an amplitude proportional to $\sin(\phi/2)$, directly determining the relative weights of the lossless and lossy paths in the subsequent evolution at MZI_2.

4.2 - Two-photon NOON-state input.

The two-photon input state used in the experiment is the path-encoded NOON state

$$|\Psi_{\mathrm{NOON}}\rangle = \frac{1}{\sqrt{2}}\left(e^{i2\phi}\,|20\rangle - |02\rangle\right), \tag{52}$$

where $|20\rangle$ and $|02\rangle$ denote two photons in the upper or lower input mode, respectively.

Action of $\mathbf{MZI_1}$. — The 2-photon input arm fock states in terms of the input arm creation $(\hat{a}_1^\dagger, \hat{a}_2^\dagger)$ are defined as follows:

$$|20\rangle_a = \frac{(\hat{a}_1^\dagger)^2}{\sqrt{2}}\,|\mathrm{vac}\rangle\,, \qquad |02\rangle_a = \frac{(\hat{a}_2^\dagger)^2}{\sqrt{2}}\,|\mathrm{vac}\rangle\,, \tag{53}$$

The action of MZI_1 on the modes is described by the same operator relations as in Sec. S4.1, namely Eqs. (44)–(47). Using the expressions for $\hat{a}_1^\dagger$ and $\hat{a}_2^\dagger$ in terms of $(\hat{b}_1^\dagger, \hat{b}_2^\dagger)$ [Eq. (47)], and substituting into Eq. (52). we obtain,

$$\begin{aligned} |\Psi_{\mathrm{NOON}}\rangle_{\mathrm{post\ MZI_1}} &= \frac{1}{\sqrt{2}}\left(e^{i2\phi}\,\frac{(\hat{a}_1^\dagger)^2}{\sqrt{2}} - \frac{(\hat{a}_2^\dagger)^2}{\sqrt{2}}\right)|\mathrm{vac}\rangle \\ &= \frac{e^{i3\pi/2}}{4}\left[\left(e^{i2\phi}-1\right)\left((\hat{b}_1^\dagger)^2+(\hat{b}_2^\dagger)^2\right)+\left(e^{i2\phi}+1\right)\left(\hat{b}_1^\dagger\hat{b}_2^\dagger+\hat{b}_2^\dagger\hat{b}_1^\dagger\right)\right]|\mathrm{vac}\rangle\,. \end{aligned} \tag{54}$$

Post–MZI_1 state in the b-arm Fock basis.— Introducing the two-photon Fock states in the arms after MZI_1,

$$|20\rangle_b = \frac{(\hat{b}_1^\dagger)^2}{\sqrt{2}}\,|\mathrm{vac}\rangle\,, \quad |02\rangle_b = \frac{(\hat{b}_2^\dagger)^2}{\sqrt{2}}\,|\mathrm{vac}\rangle\,, \quad |11\rangle_b = \hat{b}_1^\dagger\hat{b}_2^\dagger\,|{=}\rangle\,\hat{b}_2^\dagger\hat{b}_1^\dagger\,|\mathrm{vac}\rangle\,, \tag{55}$$

and the symmetric two-photon superposition

$$|2_+\rangle_b = \frac{|20\rangle_b + |02\rangle_b}{\sqrt{2}}, \tag{56}$$

Eq. (54) can be rewritten as

$$\boxed{|\Psi_{\mathrm{NOON}}\rangle_{\mathrm{post\ MZI}_1} = e^{i(3\pi/2+\phi)}\Big[\cos\phi\ |11\rangle_b + i\ \sin\phi\ |2_+\rangle_b\Big].} \tag{57}$$

Thus, after MZI_1, the NOON state is coherently distributed between the symmetric two-photon mode $|2_+\rangle_b$ (both photons in the same arm superposition) and the one-photon-per-arm configuration $|11\rangle_b$, with weights controlled by the input phase ϕ. The photon population residing in the lower b arm couples to the ancilla loss mode at MZI_2. So the programmed absorption $|A|^2$ together with the input phase ϕ controls the relative likelihood of single-photon versus two-photon absorption events. We never get perfect 2-photon absorption.

4.3 - General discrete-variable quantum state with arbitrary N.

The single- and two-photon results correspond to $N = 1$ and $N = 2$ instances of the general formalism for discrete-variable coherent absorption. An N-photon NOON state can be expanded in a basis of orthogonal effective modes with photon-number amplitudes distributed according to

$$|\mathrm{NOON}\rangle_N = 2^{-\frac{N-1}{2}} \sum_{m=0}^{N} \sqrt{\binom{N}{m}}\ \cos\left(\frac{\pi m + \phi}{2}\right) |N-m\rangle_{\mathrm{bright}}\, |m\rangle_{\mathrm{dark}}\,, \tag{58}$$

where $|N-m\rangle_{\mathrm{bright}}\,|m\rangle_{\mathrm{dark}}$ denotes a configuration with $(N-m)$ photons in the absorbing mode and m photons in the nonabsorbing mode. Equation (58) directly reproduces the discrete-variable CPA framework of Vetlugin *et al.* [3] and provides a unified description linking the single- and two-photon interference processes realized in our programmable circuit.

4.4 - Absorptivity-dependent rephasing at MZI_3 and redistribution of phase sensitivity

Here we provide an explicit analytical explanation for the different redistribution of phase sensitivity observed in the Type-1 and Type-2 lossy beamsplitters, as discussed in the main manuscript (See Main manuscript Figures 4(c,f), 5(f), and 6(f)).

The redistribution of Fisher information among the output modes is governed by the relative phase between the two fields interfering at MZI_3. From Eq. 37, the phase relation between MZI_3 and MZI_2 can be written as

$$\phi_{\mathrm{MZI}_3} - \phi_{\mathrm{MZI}_2} = \frac{\theta_{\mathrm{MZI}_2}}{2} + \frac{\pi}{2} + \mathrm{sgn}(\cos\phi_{rt})\ \tan^{-1}\left(\frac{2|t||r|\sin(\phi_{rt})}{|t|^2 - |r|^2}\right). \tag{59}$$

The relative phase between the two fields arriving at the inputs of MZI_3 is therefore

$$\Delta\phi_{\mathrm{in}} = \phi_{\mathrm{MZI}_3} - \left(\phi_{\mathrm{MZI}_2} + \frac{\theta_{\mathrm{MZI}_2}}{2} + \frac{\pi}{2}\right) = \mathrm{sgn}(\cos\phi_{rt})\ \tan^{-1}\left(\frac{2|t||r|\sin(\phi_{rt})}{|t|^2 - |r|^2}\right). \tag{60}$$

Importantly, Eq. (60) shows that all absorptivity dependence of the interference condition at MZI_3 is fully contained in the argument of the $\tan^{-1}$ term.

Since MZI_3 is programmed as a balanced (50:50) beam splitter, the effect of $\Delta\phi_{\mathrm{in}}$ on the output statistics can be made explicit. Denoting the complex field amplitudes immediately before MZI_3 by $E_1(\phi) = b_1(\phi)e^{i\beta_1}$ and $E_2(\phi) = b_2(\phi)e^{i\beta_2}$, with $\Delta\phi_{\mathrm{in}} = \beta_1 - \beta_2$, the output intensities after MZI_3 are

$$I_{\pm}(\phi) \propto \frac{1}{2}\Big[b_1^2(\phi) + b_2^2(\phi) \pm 2b_1(\phi)b_2(\phi)\sin\Delta\phi_{\mathrm{in}}\Big]. \tag{61}$$

The phase-dependent modulation, and hence the Fisher information, is therefore governed by the interference term $2b_1(\phi)b_2(\phi)\sin\Delta\phi_{\mathrm{in}}$, whose magnitude sets the fringe visibility. A fixed condition $\Delta\phi_{\mathrm{in}} = 0$ suppresses this term, leading to identical output intensities in the two signal ports, while a fixed quadrature condition $\Delta\phi_{\mathrm{in}} = \pm\pi/2$ maximizes the interference contrast and yields maximal redistribution of phase-dependent modulation between the output modes.

We emphasize that Eq. (61) describes the interference of complex field amplitudes and therefore applies directly to the single-photon measurements, where detection probabilities are proportional to output intensities. For the two-photon NOON-state experiments, the same relative phase $\Delta\phi_{\text{in}}$ governs the interference, but now through *second-order quantum interference* at MZI_3. In this case, the output probabilities are determined by the coherent superposition of two-photon probability amplitudes that acquire phase factors $\exp(\pm i\,\Delta\phi_{\text{in}})$ upon propagation to MZI_3. As a result, $\Delta\phi_{\text{in}}$ controls the visibility and phase of the two-photon oscillations (with the expected doubled phase periodicity), and therefore the redistribution of Fisher information among the output Fock states. Thus, while the microscopic interference mechanism differs between the single-photon and NOON-state cases, the role of $\Delta\phi_{\text{in}}$ as the parameter setting the effective interference condition at MZI_3 is common to both.

Type-1 mapping (fixed ϕ_{rt}): In the Type-1 configuration, the internal phase is fixed to $\phi_{rt} = \pi$, while Eqs. 2 enforce a change in the ratio $|t|/|r|$ as the absorption probability $|A|^2$ is varied. Substituting $\phi_{rt} = \pi$ into the argument of the $\tan^{-1}$ term in Eq. (60) yields

$$\operatorname{sgn}(\cos\phi_{rt})\ \tan^{-1}\left(\frac{2|t||r|\sin(\phi_{rt})}{|t|^2 - |r|^2}\right) = 0, \tag{62}$$

so that the relative phase between the two fields arriving at MZI_3 is fixed to $\Delta\phi_{\text{in}} = 0$, independent of $|A|^2$. Accordingly, tuning $|A|^2$ does not alter the phase condition of the interference at MZI_3, but instead rescales the relative amplitudes of the fields entering it. In particular, the amplitude reaching MZI_3 from the lower arm (mode 2) is reduced as $|A|^2$ increases, while its phase relation with the upper arm remains unchanged. As a result, the output-intensity fringes in the two signal ports remain identical and exhibit a uniform reduction in visibility, leading to a simultaneous suppression of phase sensitivity across all output modes as $|A|^2$ is increased.

Type-2 mapping ($|t| = |r|$): In the Type-2 configuration, Eqs. 2 impose $|t| = |r| = \sqrt{(1-|A|^2)/2}$, while the internal phase ϕ_{rt} becomes a function of $|A|^2$. Substituting $|t| = |r|$ into Eq. (60) gives

$$\operatorname{sgn}(\cos\phi_{rt})\,\tan^{-1}\!\left(\frac{2|t||r|\sin(\phi_{rt})}{|t|^2-|r|^2}\right) \;\to\; \pm\infty, \tag{63}$$

so that

$$\Delta\phi_{\text{in}} = \pm\frac{\pi}{2} \pmod{\pi}, \tag{64}$$

with the sign determined by $\sin\phi_{rt}$. Thus, in the Type-2 mapping the two fields interfere at MZI_3 under a fixed quadrature condition. As in the Type-1 case, varying $|A|^2$ does not modify the interference phase itself, but instead changes the relative weighting of the amplitudes $a_{1,2}(\phi)$ arriving at MZI_3. However, because this rescaling acts at $\Delta\phi_{\text{in}} = \pm\pi/2$, it leads to a redistribution of phase-dependent modulation among the output modes, and consequently to the observed transfer of phase sensitivity from the ancilla to the signal ports as $|A|^2$ is reduced.

In summary, in both mappings the absorption parameter $|A|^2$ tunes the relative amplitudes entering the second interference stage without rephasing the fields at MZI_3. The qualitative difference between the two cases arises from the fixed value of the relative phase $\Delta\phi_{\text{in}}$ at which this amplitude tuning occurs: $\Delta\phi_{\text{in}} = 0$ for Type-1 and $\Delta\phi_{\text{in}} = \pm\pi/2$ for Type-2. This distinction underlies the markedly different redistribution of Fisher information observed in the two mappings.

Supplementary Note 5 - Additional details on Maximum Classical Fisher information calculation.

Additional details of the Fisher information analysis for both single-photon and NOON state experiments are provided in Supplementary Figures 5-8, which show the sinusoidal fits used to extract the phase derivatives and the resulting comparisons between experimental and theoretical maximum classical Fisher information values across all output modes and $|A|^2$ settings. The experimentally obtained fisher information heatmaps show good agreement with the theoretically predicted values.

Supplementary Note 6 - Origin of the $|200\rangle$-$|020\rangle$ imbalance

NB: Throughout this section, Fock-state notation is used as follows. States written with two occupation numbers refer to the photon-number occupation of the two signal modes, ordered as (signal 1, signal 2). States written with three occupation numbers refer to the photon-number occupation of all three relevant modes, ordered as (signal 1, signal 2, ancilla).

The outcomes $|200\rangle$ and $|020\rangle$ correspond to same-mode two-photon events at the two signal outputs of the programmable photonic circuit and are identified experimentally using the photon-number-resolving (PNR) Mach-Zehnder interferometers that follow each signal port. For a given two-photon state injected into the full circuit, the measured two-photon statistics at the signal outputs are finally governed by the effective two-port transformation implemented by the final interferometric stage (MZI_3).

For an ideal symmetric two-port transformation at MZI_3, the probabilities of observing $|200\rangle$ and $|020\rangle$ are expected to be equal whenever these channels are populated. Experimentally, we observe a systematic $|200\rangle$-$|020\rangle$ imbalance that is most pronounced at low $|A|^2$ values and becomes negligible close to $|A|^2 = 0.5$. Below we (i) map the externally prepared circuit in-

put state to the effective state incident on MZI_3, and (ii) show how a single residual amplitude asymmetry in the effective two-port scattering coefficients of MZI_3 reproduces the key observations: the same imbalance magnitude for both symmetric inputs ($|11\rangle$ and symmetric NOON) to MZI_3, and a near-ideal response for the antisymmetric NOON input to MZI_3.

6.1 - Mapping from circuit input state to effective MZI_3 input state

The two-photon input to the circuit is the path-encoded NOON state prepared by the state-preparation MZI and defined in the first two modes as

$$|\Psi_{\mathrm{NOON}}\rangle = \frac{1}{\sqrt{2}}\Big(e^{i2\phi}|20\rangle - |02\rangle\Big), \tag{65}$$

where the experimentally scanned parameter ϕ sets the relative phase between the two bunched components.

In the discussion below, we first consider the zero-absorption limit ($|A|^2 = 0$), for which no population is routed to the ancilla mode and the entire optical field exiting the lower arm of MZI_1 is incident on MZI_3.

After the first interference stage (MZI_1), the state in the arm basis (b_1, b_2) becomes

$$|\Psi_{\mathrm{NOON}}\rangle_{\mathrm{post\ MZI1}} = e^{i(3\pi/2+\phi)}\Big[\cos(\phi)\,|11\rangle_b + i\sin(\phi)\,|2+\rangle_b\Big], \tag{66}$$

with $|2+\rangle_b = (|20\rangle_b + |02\rangle_b)/\sqrt{2}$. Thus, $\phi = 0, \pi, \ldots$ yields $|11\rangle_b$, while $\phi = \pi/2, 3\pi/2, \ldots$ yields the symmetric NOON state $|2+\rangle_b$ (up to a global phase).

Immediately before MZI_3, a fixed relative phase $\Delta\phi$ dictated by Eq. 37 is applied between the two arms, defining the two beamsplitter classes:

- **Type-1 beamsplitter:** $\Delta\phi = 0$,
- **Type-2 beamsplitter:** $\Delta\phi = \pi/2$.

For the Type-1 setting, the state in Eq. (66) enters MZI_3 unchanged. For the Type-2 setting, the applied $\pi/2$ phase converts the symmetric NOON component into the antisymmetric NOON state,

$$\frac{|20\rangle + |02\rangle}{\sqrt{2}} \longrightarrow \frac{|20\rangle - |02\rangle}{\sqrt{2}} \quad \text{(up to a global phase)}, \tag{67}$$

while leaving the $|11\rangle$ component invariant. Depending on ϕ and on the beamsplitter type, the effective two-photon state incident on MZI_3 is therefore $|11\rangle$, a symmetric NOON state, an antisymmetric NOON state, or a coherent superposition thereof.

This mapping exhaustively describes the input states to MZI_3 only in the zero-absorption limit, in which all two-photon components exiting the lower arm of MZI_1 reach MZI_3 without routing to the ancilla mode. At finite absorption ($|A|^2 > 0$), a fraction of the optical field is coherently routed to the ancilla mode. As $|A|^2$ increases, the weight of two-photon *symmetric* components incident on MZI_3 (i.e., $|11\rangle$ and symmetric NOON) decreases, while the relative contribution of effective single-photon or bunched-with-ancilla inputs (e.g. $|10\rangle$ and $|20\rangle$ in the signal subsystem) increases. Consistent with this redistribution, we observe that deviations from ideal symmetric two-photon interference are largest at low $|A|^2$ and progressively diminish as $|A|^2 \to 0.5$.

6.2 - Effective two-port description motivated by the measured MZI_3 output statistics

The measured two-photon statistics reveal three systematic features that depend on the effective state incident on MZI_3: (i) for a single-photon input to MZI_3 ($|10\rangle$), the two outputs are balanced; (ii) for symmetric two-photon inputs to MZI_3 ($|11\rangle$ and symmetric NOON), the $|200\rangle$ and $|020\rangle$ outcomes are imbalanced with essentially the same magnitude; and (iii) for an antisymmetric NOON input to MZI_3, the output remains concentrated in the $|11\rangle$ channel with strongly suppressed bunched terms.

To account for these observations, we describe MZI_3 by a general linear two-port scattering matrix

$$S_{\mathrm{MZI}_3} = \begin{pmatrix} t & r \\ r' & t' \end{pmatrix}, \tag{68}$$

which characterizes the transformation experienced by the two signal modes incident on MZI_3

All MZIs on the chip, including MZI_3, are calibrated using classical coherent light injected into a single input port (the upper input port in our implementation for MZI_3), and the heater phases are adjusted such that the output powers are balanced for that calibration configuration[1]. This imposes the first-order constraint

$$|t|^2 = |r'|^2 = \frac{1}{2}, \tag{69}$$

but it does not uniquely determine the remaining amplitudes r and t' nor the coherent phase relations between $\{t, r, r', t'\}$ that enter two-photon interference.

To capture the experimentally observed $|200\rangle$-$|020\rangle$ imbalance within this freedom, we introduce a single real parameter λ that quantifies a residual amplitude asymmetry between the two complementary scattering paths of MZI_3. In the analysis below we therefore choose the following complex coefficients for the effective two-port transformation:

$$t = \frac{1}{\sqrt{2}}, \quad r = \frac{\lambda}{\sqrt{2}}, \quad r' = \frac{1}{\sqrt{2}}, \quad t' = -\frac{\lambda^{-1}}{\sqrt{2}}, \tag{70}$$

where $\lambda = 1$ corresponds to the ideal symmetric case, and $\lambda \neq 1$ produces unequal bunched two-photon amplitudes while remaining fully consistent with the single-photon calibration constraint of Eq. (69).

6.3 - Two-photon transformation motivated by a $|11\rangle$ input to MZI_3

We first consider the case in which the effective input to MZI_3 is the two-photon state $|11\rangle$. Experimentally, this configuration exhibits a systematic imbalance between the $|200\rangle$ and $|020\rangle$ outcomes.

The transformation of a $|11\rangle$ input to MZI_3 is

$$|11\rangle_{\mathrm{MZI}_3} \longrightarrow \sqrt{2}\,tr\,|20\rangle + (tt' + rr')\,|11\rangle + \sqrt{2}\,r't'\,|02\rangle. \tag{71}$$

The corresponding bunched-output probabilities satisfy

$$P_{20}^{(11)} \propto |tr|^2, \qquad P_{02}^{(11)} \propto |r't'|^2. \tag{72}$$

Therefore, the observed $|200\rangle$-$|020\rangle$ imbalance directly implies

$$|tr| \neq |r't'|, \tag{73}$$

i.e., an effective asymmetry in the scattering amplitudes of MZI_3 that is not fixed by calibration based on first-order (classical) transmission measurements.

6.4 - Extension to symmetric NOON inputs incident on MZI_3

We next consider the case where the effective input to MZI_3 is the symmetric NOON state $|\mathrm{NOON}_+\rangle = (|20\rangle + |02\rangle)/\sqrt{2}$. Experimentally, this configuration exhibits a $|200\rangle$-$|020\rangle$ imbalance of essentially the same magnitude as observed for the $|11\rangle$ input.

The corresponding transformation is

$$|\mathrm{NOON}_+\rangle_{\mathrm{MZI}_3} \longrightarrow \frac{t^2 + r^2}{\sqrt{2}}\,|20\rangle + (tr' + rt')\,|11\rangle + \frac{r'^2 + t'^2}{\sqrt{2}}\,|02\rangle. \tag{74}$$

Thus, the bunched-output probabilities are governed by $|t^2 + r^2|^2$ and $|r'^2 + t'^2|^2$, which depend on the same scattering amplitudes $\{t, r, r', t'\}$ as in the $|11\rangle$ case. In addition, the $|11\rangle$ output for the symmetric NOON input is controlled by the coherent sum $(tr' + rt')$. In the ideal case this term cancels, whereas residual phase and amplitude imperfections generically yield a small but finite $|11\rangle$ population, consistent with our measurements near operating points where this channel is ideally suppressed.

6.5 - Identical imbalance for symmetric inputs to MZI_3

To capture the experimentally observed fact that the *magnitude* of the $|200\rangle$-$|020\rangle$ imbalance is essentially the same whenever the input to MZI_3 is symmetric (i.e., for both $|11\rangle$ and symmetric NOON), it is sufficient to retain only the first-order balance constraint of Eq. (69) and allow a residual asymmetry in the remaining amplitudes. We therefore parameterize

$$|t| = |r'| = \sqrt{\tfrac{1}{2}}, \qquad |r| = \sqrt{\tfrac{1}{2}}\,\lambda, \qquad |t'| = \sqrt{\tfrac{1}{2}}\,\lambda^{-1}, \tag{75}$$

where $\lambda = 1$ corresponds to the ideal symmetric case.

With Eq. (75), the bunched-output probabilities for a $|11\rangle$ input obey

$$P_{20}^{(11)} \propto |tr|^2 = \tfrac{1}{4}\lambda^2, \qquad P_{02}^{(11)} \propto |r't'|^2 = \tfrac{1}{4}\lambda^{-2}, \tag{76}$$

so that

$$\frac{P_{20}^{(11)}}{P_{02}^{(11)}} = \lambda^4. \tag{77}$$

For a symmetric NOON input $|\mathrm{NOON}_+\rangle = (|20\rangle + |02\rangle)/\sqrt{2}$, the bunched output amplitudes are given by Eq. (74),

$$A_{20}^{(+)} = \frac{t^2 + r^2}{\sqrt{2}}, \qquad A_{02}^{(+)} = \frac{r'^2 + t'^2}{\sqrt{2}}.$$

Using Eq. (75), the corresponding bunched probabilities are

$$P_{20}^{(+)} \propto |t^2 + r^2|^2 = \tfrac{1}{4}\left(1 + \lambda^2\right)^2, \qquad P_{02}^{(+)} \propto |r'^2 + t'^2|^2 = \tfrac{1}{4}\left(1 + \lambda^{-2}\right)^2, \tag{78}$$

yielding the exact ratio

$$\frac{P_{20}^{(+)}}{P_{02}^{(+)}} = \left(\frac{1 + \lambda^2}{1 + \lambda^{-2}}\right)^2. \tag{79}$$

Although Eqs. (77) and (79) are not identical, their difference enters only at second order in the residual amplitude asymmetry, so that the resulting $|200\rangle$-$|020\rangle$ imbalance is of comparable magnitude for both symmetric inputs.

To quantify the imbalance we introduce the normalized ratio

$$\mathcal{I} = \frac{P_{20} - P_{02}}{P_{20} + P_{02}}. \tag{80}$$

For the $|11\rangle$ input, Eq. (76) yields

$$\mathcal{I}^{(11)} = \frac{\lambda^2 - \lambda^{-2}}{\lambda^2 + \lambda^{-2}} = \frac{\lambda^4 - 1}{\lambda^4 + 1}. \tag{81}$$

Using the largest deviation observed in our measurements (approximately a 20% imbalance at the lowest $|A|^2$ values), i.e. $\mathcal{I}^{(11)} \approx 0.2$, Eq. (81) implies $\lambda^4 = (1+\mathcal{I})/(1-\mathcal{I}) \approx 1.5$ and therefore

$$\lambda \approx 1.5^{1/4} \approx 1.11, \qquad \lambda^2 \approx 1.23, \qquad \lambda^{-2} \approx 0.81. \tag{82}$$

Thus, a $\sim 20\%$ imbalance in the bunched probabilities corresponds to only a $\sim 10\%$ asymmetry in the underlying scattering amplitudes.

Output-state coefficients for symmetric inputs. We evaluate the normalized output-state coefficients for the two symmetric effective inputs to MZI_3. While the $|11\rangle$ component depends on coherent sums of scattering amplitudes, the relative weights of the bunched components are fixed by the amplitude asymmetry parameter λ.

(i) $|11\rangle$ input. From Eq. (71), the output amplitudes are

$$A_{20}^{(11)} = \sqrt{2}\,tr, \qquad A_{11}^{(11)} = tt' + rr', \qquad A_{02}^{(11)} = \sqrt{2}\,r't'. \tag{83}$$

Substituting Eq. (70) gives,

$$A_{20}^{(11)} = \frac{\lambda}{\sqrt{2}}, \qquad A_{11}^{(11)} = \frac{\lambda - \lambda^{-1}}{2}, \qquad A_{02}^{(11)} = -\frac{\lambda^{-1}}{\sqrt{2}}. \tag{84}$$

The normalization factor

$$\mathcal{N}_{11}^2 = \frac{\lambda^2 + \lambda^{-2}}{2} + \frac{(\lambda - \lambda^{-1})^2}{4} \tag{85}$$

yields the normalized coefficients

$$\left(c_{20}^{(11)}, c_{11}^{(11)}, c_{02}^{(11)}\right) = \frac{1}{\mathcal{N}_{11}} \left(\frac{\lambda}{\sqrt{2}}, \ \frac{\lambda - \lambda^{-1}}{2}, \ -\frac{\lambda^{-1}}{\sqrt{2}} \right). \tag{86}$$

For $\lambda \simeq 1.11$, this corresponds to probabilities $(|c_{20}^{(11)}|^2, |c_{11}^{(11)}|^2, |c_{02}^{(11)}|^2) \approx (0.60, 0.01, 0.39)$.

(ii) Symmetric NOON input. From Eq. (74), the bunched amplitudes are

$$A_{20}^{(+)} = \frac{t^2 + r^2}{\sqrt{2}}, \qquad A_{02}^{(+)} = \frac{r'^2 + t'^2}{\sqrt{2}}, \tag{87}$$

while the $|11\rangle$ term vanishes. Substituting Eq. (70) gives

$$A_{20}^{(+)} = \frac{1 + \lambda^2}{2\sqrt{2}}, \qquad A_{02}^{(+)} = \frac{1 + \lambda^{-2}}{2\sqrt{2}}. \tag{88}$$

With normalization

$$\mathcal{N}_+^2 = \frac{(1 + \lambda^2)^2 + (1 + \lambda^{-2})^2}{8}, \tag{89}$$

the normalized coefficients are

$$\left(c_{20}^{(+)}, c_{11}^{(+)}, c_{02}^{(+)}\right) = \frac{1}{\mathcal{N}_+} \left(\frac{1 + \lambda^2}{2\sqrt{2}}, \ 0, \ \frac{1 + \lambda^{-2}}{2\sqrt{2}} \right). \tag{90}$$

For $\lambda \simeq 1.11$, this gives bunched probabilities $(|c_{20}^{(+)}|^2, |c_{02}^{(+)}|^2) \approx (0.60, 0.40)$, matching the imbalance magnitude of the $|11\rangle$ input case.

6.6 - Antisymmetric NOON input to MZI$_3$

For an antisymmetric NOON input $|\text{NOON}_-\rangle = (|20\rangle - |02\rangle)/\sqrt{2}$, the output state is

$$|\text{NOON}_-\rangle_{\text{out}} = \frac{t^2 - r^2}{\sqrt{2}} |20\rangle + (tr' - rt') |11\rangle + \frac{r'^2 - t'^2}{\sqrt{2}} |02\rangle. \tag{91}$$

Substituting Eq. (70) gives

$$A_{20}^{(-)} = \frac{1 - \lambda^2}{2\sqrt{2}}, \qquad A_{02}^{(-)} = \frac{1 - \lambda^{-2}}{2\sqrt{2}}, \qquad A_{11}^{(-)} = 1 \tag{92}$$

(up to a global phase). For $\lambda \simeq 1.11$, the bunched probabilities are

$$P_{20}^{(-)} \approx 6.7 \times 10^{-3}, \qquad P_{02}^{(-)} \approx 4.5 \times 10^{-3}, \tag{93}$$

i.e. both are suppressed at the sub-percent level.

The normalized output coefficients follow from

$$\mathcal{N}_-^2 = 1 + \frac{(1-\lambda^2)^2 + (1-\lambda^{-2})^2}{8}, \tag{94}$$

yielding

$$\left(c_{20}^{(-)}, c_{11}^{(-)}, c_{02}^{(-)}\right) = \frac{1}{\mathcal{N}_-}\left(\frac{1-\lambda^2}{2\sqrt{2}},\ e^{i(\varphi_t+\varphi_{r'})},\ \frac{1-\lambda^{-2}}{2\sqrt{2}}\right). \tag{95}$$

For $\lambda \simeq 1.11$, the output is $\approx 99\%$ in the $|11\rangle$ channel, consistent with the observed near-ideal antisymmetric NOON response.

Comparison to the full NOON-state dataset. To explicitly validate that the single-parameter MZI3-asymmetry model introduced in Eq. (S70) accounts for the experimentally observed deviations across the entire two-photon dataset, we overlay the measured NOON-state output statistics with the corresponding theoretical curves computed using the same effective two-port description of MZI_3 as in Eqs. 71, 74, and 91. Supplementary Figures 9 and 10 show the measured probabilities for all six detected Fock outcomes as a function of the scanned NOON phase ϕ, for all programmed $|A|^2$ values, together with the theoretical predictions that include the residual amplitude asymmetry parameter λ in the transfer matrix of MZI_3. Overall, for $\lambda = 1.10$ (consistent with the estimate from the maximal low-$|A|^2$, the experimental data shows good agreement with the modified theoretical curves that explicitly include the residual MZI_3 asymmetry, thereby supporting our interpretation of the observed deviations within the framework developed in Section S6.

6.7 - Physical origin of the residual asymmetry in MZI$_3$ and implications for calibration

The residual $|200\rangle$-$|020\rangle$ imbalance discussed above indicates a small asymmetry in the effective 2×2 transfer matrix of MZI$_3$. Plausible physical origins of such an asymmetry include fabrication-induced imbalance of the MMIs, differential propagation loss between the two interferometer arms and weak parasitic reflections or mode mismatch at waveguide junctions. Any of these effects can break the ideal port symmetry of the MZI and give rise to unequal amplitude products such as $|tr| \neq |r't'|$, while remaining largely invisible to first-order transmission measurements.

In our implementation, all MZIs were calibrated using classical coherent light injected into a single input port[1]. By sweeping the internal phase θ of each MZI and measuring the corresponding output power splitting ratio, we established a mapping between heater bias and the effective splitting ratio of the interferometer. This procedure correctly fixes the magnitude of the scattering coefficients associated with the excited input port, but it does not determine the full complex 2×2 scattering matrix. In particular, single-port, intensity-only calibration constrains only a subset of the effective scattering parameters [Eq. (69)], while leaving undetermined the relative phases and amplitude products that govern two-photon interference. By contrast, two-photon interference directly probes coherent combinations of the scattering coefficients, such as tr, $r't'$, $t^2 + r^2$, and $tr' + rt'$, which are not fixed by such a calibration procedure [Eqs. (71) and (74)].

Single-port classical calibration is sufficient for programmable MZI meshes under the assumption of port-symmetric interferometers, which is the operating model used in standard Clements and Reck architectures. However, in the present device, the MZIs employ a single internal phase shifter and therefore cannot independently control all parameters of a general 2×2 scattering matrix. As a result, deviations from ideal port symmetry caused by fabrication

imperfections cannot, in general, be fully corrected by phase tuning alone, even if both input ports are characterized classically. The accessible set of effective transformations is therefore restricted, and certain asymmetries that are invisible to single-port calibration can manifest in two-photon interference.

A complete suppression of the residual imbalance would require either improved fabrication uniformity to restore near-ideal port symmetry, or an extended MZI design providing additional phase-shifting degrees of freedom, together with a calibration protocol that constrains the full complex scattering matrix. While calibration using quantum light can directly access the relevant two-photon interference parameters, such approaches do not scale efficiently to large interferometric meshes due to the limited brightness of quantum light sources.

These considerations clarify that the observed $|200\rangle$-$|020\rangle$ imbalance arises from a small, physically plausible residual asymmetry of the MZI transfer matrix that remains compatible with standard classical calibration procedures, and does not indicate a breakdown of the overall interferometric control of the circuit.

Supplementary Note 7 - Effect of photon distinguishability on two-photon interferometric signatures

In this section, we theoretically quantify how photon distinguishability affects phase-dependent two-photon output statistics, coherent absorption signatures, and the associated Fisher information (FI). The analysis is performed within the same device model used throughout this work, while explicitly accounting for the fact that distinguishable photons do not interfere coherently.

7.1 - Model of partial distinguishability

Photon distinguishability is parametrized by $d \in [0, 1]$, where $d = 0$ corresponds to perfectly indistinguishable photons and $d = 1$ to fully distinguishable photons. For a given two-photon

output Fock state $|k\rangle$, the detection probability is expressed as a convex mixture

$$P_k(\phi; d) = (1 - d)\, P_k^{\mathrm{ind}}(\phi) + d\, P_k^{\mathrm{dist}}, \tag{96}$$

where $P_k^{\mathrm{ind}}(\phi)$ denotes the phase-dependent probability arising from two-photon interference, and P_k^{dist} denotes the corresponding probability for fully distinguishable photons. This decomposition reflects the absence of two-photon interference in the fully distinguishable limit.

In the experiment, the two-photon NOON state is prepared via Hong-Ou-Mandel interference at the state-preparation stage. Partial distinguishability reduces the weight of the interference-enabled NOON component and increases the relative contribution of non-interfering events. Equation (96) captures this behaviour directly at the level of output detection probabilities, without modifying the underlying device transformation.

7.2 - Computational method

The propagation of indistinguishable photons is treated using the same two-photon interferometric framework described in the Methods section of the main manuscript. The lossy two-port device is embedded into a larger quasiunitary scattering matrix via minimal dilation, and the corresponding two-photon transformation is constructed to evaluate $P_k^{\mathrm{ind}}(\phi)$.

For fully distinguishable photons (which do not end up forming NOON states), each photon is propagated independently through the device using the single-photon transformation associated with the same quasiunitary dilation. The resulting two-photon output probabilities P_k^{dist} are obtained by combining the independent single-photon probabilities in a purely probabilistic manner. This approach correctly captures the behaviour of distinguishable photons, which do not interfere coherently and whose joint detection statistics arise from independent single-photon events.

Photon distinguishability is introduced solely at the input-state level and does not modify

the device scattering matrix, the quasiunitary dilation, or the underlying single-photon transformation.

7.3 - Phase-dependent output probabilities

The phase-dependent two-photon output probabilities for varying distinguishability are shown in Supplementary Figures 11 and 12 for the π-phase-shifted (Type 1) and symmetric (Type 2) beamsplitter implementations, respectively. Results are shown for several distinguishability values and for two limiting absorption regimes: a lossless device ($|A|^2 = 0$) and a strongly absorbing device ($|A|^2 = 0.5$).

In both beamsplitter implementations, increasing photon distinguishability leads to a continuous suppression of the phase-dependent modulation across all two-photon output channels. In the fully distinguishable limit ($d = 1$), the output probabilities become ϕ-independent. In the absorbing case ($|A|^2 = 0.5$), absorption-related output channels remain populated even at $d = 1$, indicating that absorption persists while losing its phase sensitivity.

7.4 - Fisher information

From the calculated output probabilities, we evaluate the total Fisher information for estimation of the NOON phase ϕ,

$$F_{\mathrm{tot}}(\phi) = \sum_i \frac{1}{P_i(\phi)} \left(\frac{dP_i(\phi)}{d\phi} \right)^2 , \tag{97}$$

where the index i runs over the six two-photon output Fock basis states.

Supplementary Figure 13(a,b) show the Fisher information as a function of ϕ for different distinguishability values for the Type 1 and Type 2 beamsplitters, respectively. Supplementary Figure 13(c) shows the maximum Fisher information, optimized over ϕ, as a function of d.

For both beamsplitter implementations, the maximum Fisher information decreases with increasing distinguishability and vanishes in the fully distinguishable limit, confirming that pho-

ton indistinguishability is essential for phase sensitivity in the present scheme.

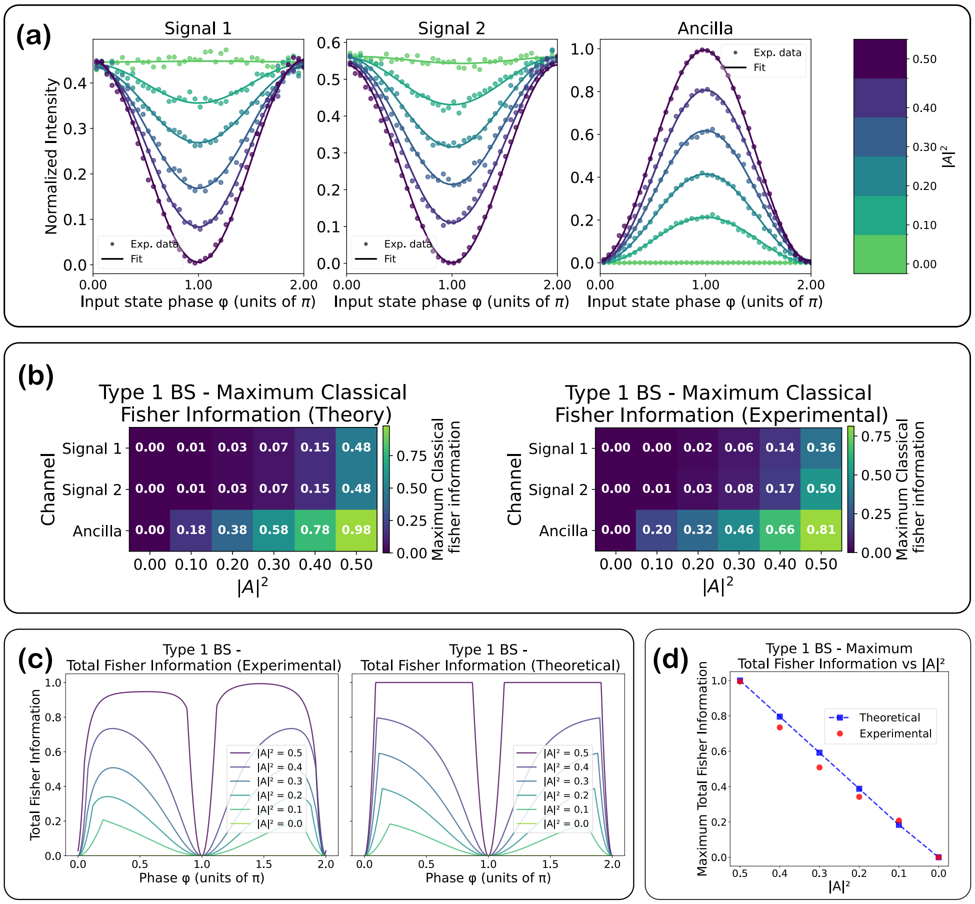

Supplementary Figure 5: **Fisher information analysis for the single-photon experiment (Type 1 configuration).** (a) Measured single-photon output probabilities (markers) and sinusoidal fits (solid lines) versus the input-state phase ϕ (in units of π) for the three monitored output modes (Signal 1, Signal 2, and Ancilla), shown for multiple programmed absorptivities $|A|^2$ (color-coded). (b) Heatmaps of the *maximum* classical Fisher information extracted for each output mode as a function of $|A|^2$, shown for the theoretical model (left) and experiment (right). (c) Total Fisher information (summed over all output modes) versus ϕ for several $|A|^2$ values, shown for experiment (left) and theory (right). (d) Maximum total Fisher information versus $|A|^2$, comparing theory (blue) and experiment (red).

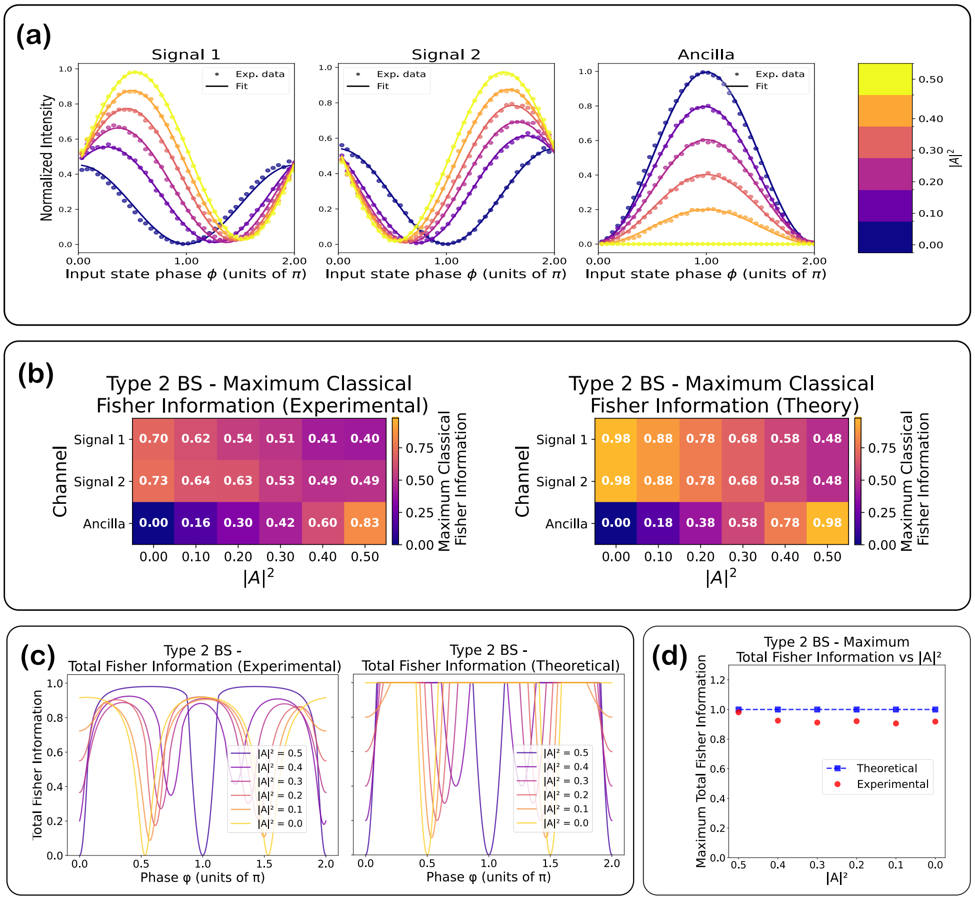

Supplementary Figure 6: **Fisher information analysis for the single-photon experiment (Type 2 configuration).** (a) Measured single-photon output probabilities (markers) and sinusoidal fits (solid lines) versus the input-state phase ϕ (in units of π) for Signal 1, Signal 2, and Ancilla, shown for multiple programmed absorptivities $|A|^2$ (color-coded). (b) Heatmaps of the *maximum* classical Fisher information extracted for each output mode as a function of $|A|^2$, shown for experiment (left) and the theoretical model (right). (c) Total Fisher information (summed over all output modes) versus ϕ for several $|A|^2$ values, shown for experiment (left) and theory (right). (d) Maximum total Fisher information versus $|A|^2$, comparing theory (blue) and experiment (red).

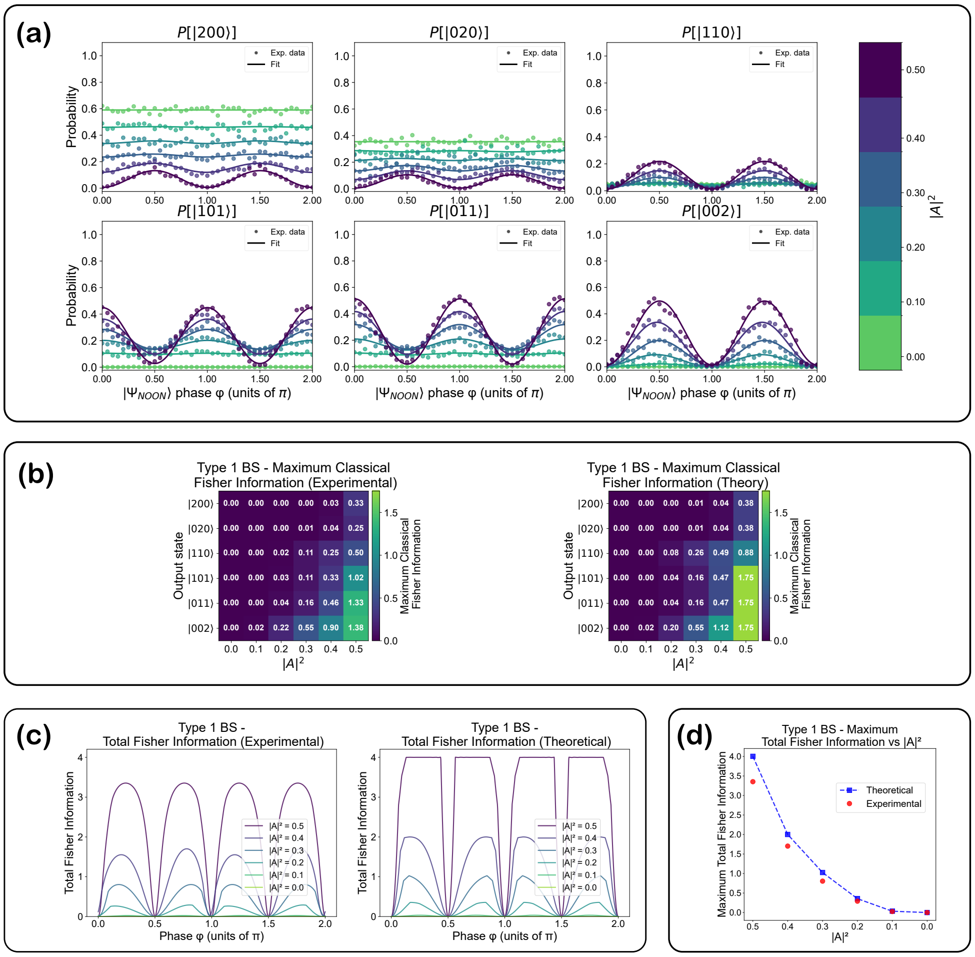

Supplementary Figure 7: **Fisher information analysis for the NOON-state experiment (Type 1 configuration).** (a) Measured two-photon output probabilities (markers) and sinusoidal fits (solid lines) versus the NOON-state phase ϕ (in units of π), shown for the six detected outcomes $|200\rangle$, $|020\rangle$, $|110\rangle$, $|101\rangle$, $|011\rangle$, and $|002\rangle$, and for multiple programmed absorptivities $|A|^2$ (color-coded). (b) Heatmaps of the *maximum* classical Fisher information extracted for each detected outcome as a function of $|A|^2$, shown for experiment (left) and the theoretical model (right). (c) Total Fisher information (summed over all detected outcomes) versus ϕ for several $|A|^2$ values, shown for experiment (left) and theory (right). (d) Maximum total Fisher information versus $|A|^2$, comparing theory (blue) and experiment (red).

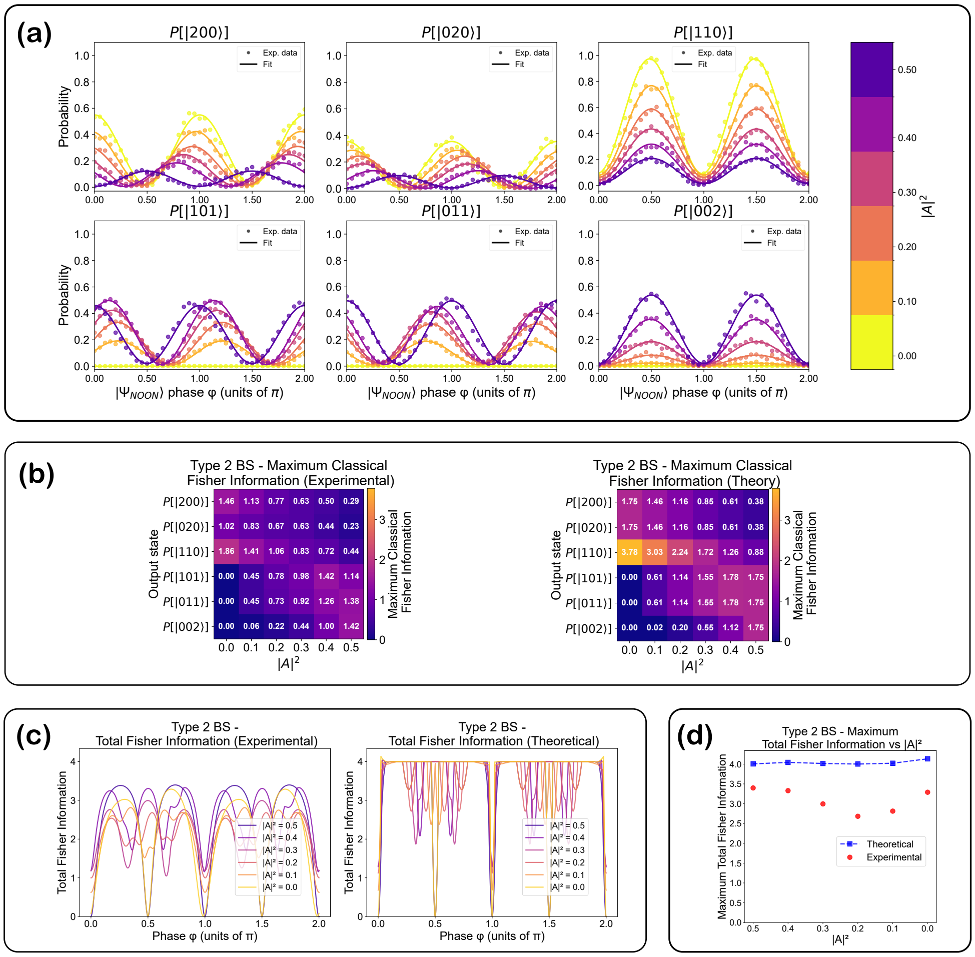

Supplementary Figure 8: **Fisher information analysis for the NOON-state experiment (Type 2 configuration).** (a) Measured two-photon output probabilities (markers) and sinusoidal fits (solid lines) versus the NOON-state phase ϕ (in units of π), shown for the six detected outcomes $|200\rangle$, $|020\rangle$, $|110\rangle$, $|101\rangle$, $|011\rangle$, and $|002\rangle$, and for multiple programmed absorptivities $|A|^2$ (color-coded). (b) Heatmaps of the *maximum* classical Fisher information extracted for each detected outcome as a function of $|A|^2$, shown for experiment (left) and the theoretical model (right). (c) Total Fisher information (summed over all detected outcomes) versus ϕ for several $|A|^2$ values, shown for experiment (left) and theory (right). (d) Maximum total Fisher information versus $|A|^2$, comparing theory (blue) and experiment (red).

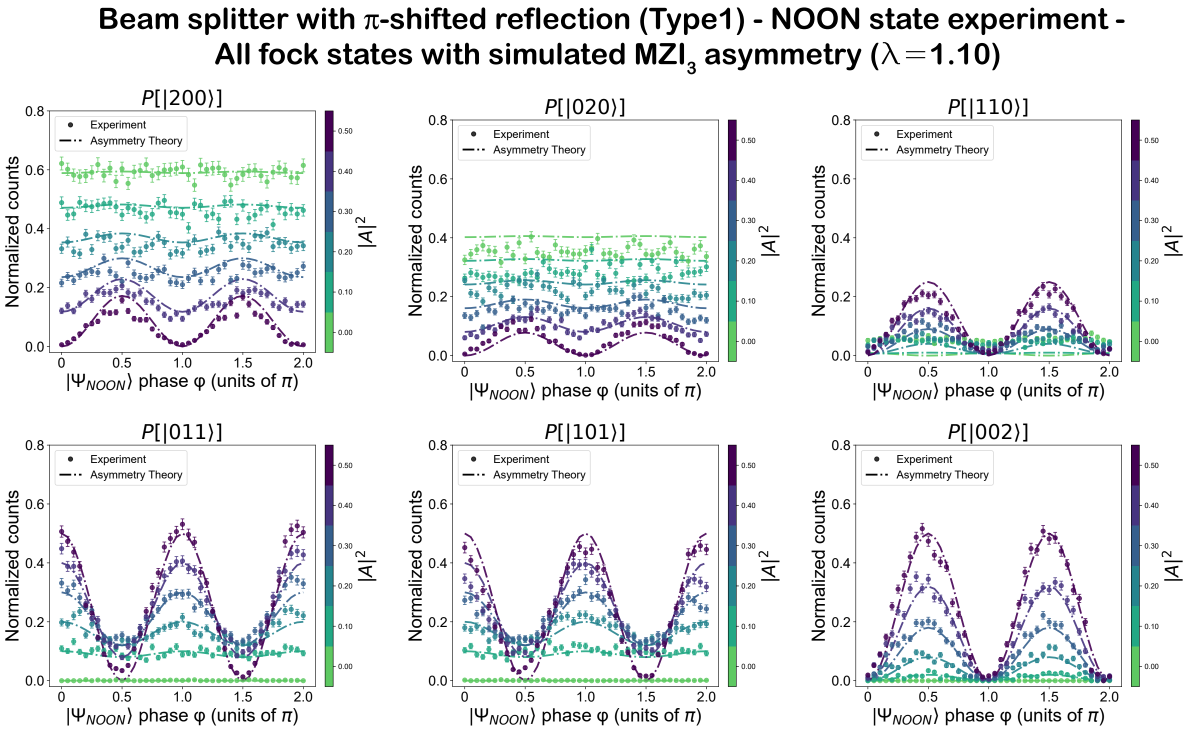

Supplementary Figure 9: **Type-1 ($\phi_{rt} = \pi$) NOON-state data with MZI3-asymmetry model overlay.** Measured two-photon output statistics (markers) for the six detected Fock outcomes $|200\rangle, |020\rangle, |110\rangle, |101\rangle, |011\rangle$, and $|002\rangle$ as a function of the scanned NOON phase ϕ (units of π), shown for all programmed $|A|^2$ values (color-coded). Error bars represent one standard deviation, calculated as $\sqrt{N}$ from Poisson counting statistics, with proper propagation through the normalization procedure. Dash-dotted curves show the corresponding theoretical prediction obtained by propagating the effective MZI_3 input state through the general two-port scattering model $S_{\mathrm{MZI}_3} = \begin{pmatrix} t & r \\ r' & t' \end{pmatrix}$ and including the residual amplitude asymmetry parameter $\lambda \neq 1$ introduced in Eq. 70. The value $\lambda = 1.10$ (consistent with Eq. 82 is used globally for all $|A|^2$ settings. The model reproduces the observed $|200\rangle$-$|020\rangle$ imbalance for symmetric effective inputs to MZI_3 while preserving the near-ideal antisymmetric-NOON response.

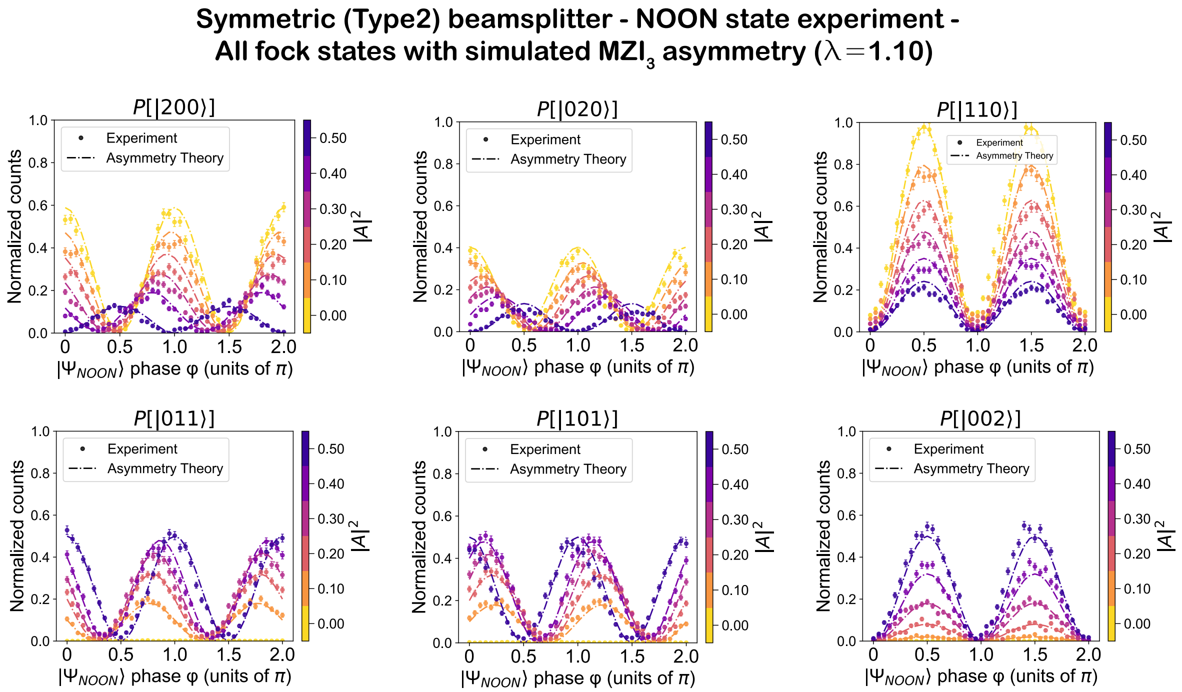

Supplementary Figure 10: **Type-2 (symmetric) NOON-state data with MZI3-asymmetry model overlay.** Same as Supplementary Figure 9, but for the Type-2 (symmetric) beamsplitter implementation. Markers show measured normalized two-photon counts versus the scanned NOON phase ϕ, for all $|A|^2$ values (color-coded). Error bars represent one standard deviation, calculated as $\sqrt{N}$ from Poisson counting statistics, with proper propagation through the normalization procedure. Dash-dotted curves show the theoretical prediction including the same effective MZI_3 scattering model and same residual amplitude asymmetry parameter $\lambda = 1.10$ [Eq. (S70)], which captures the bunched-output imbalance for symmetric effective inputs and leaves the antisymmetric-NOON response largely unchanged, consistent with Eqs. 91-95.

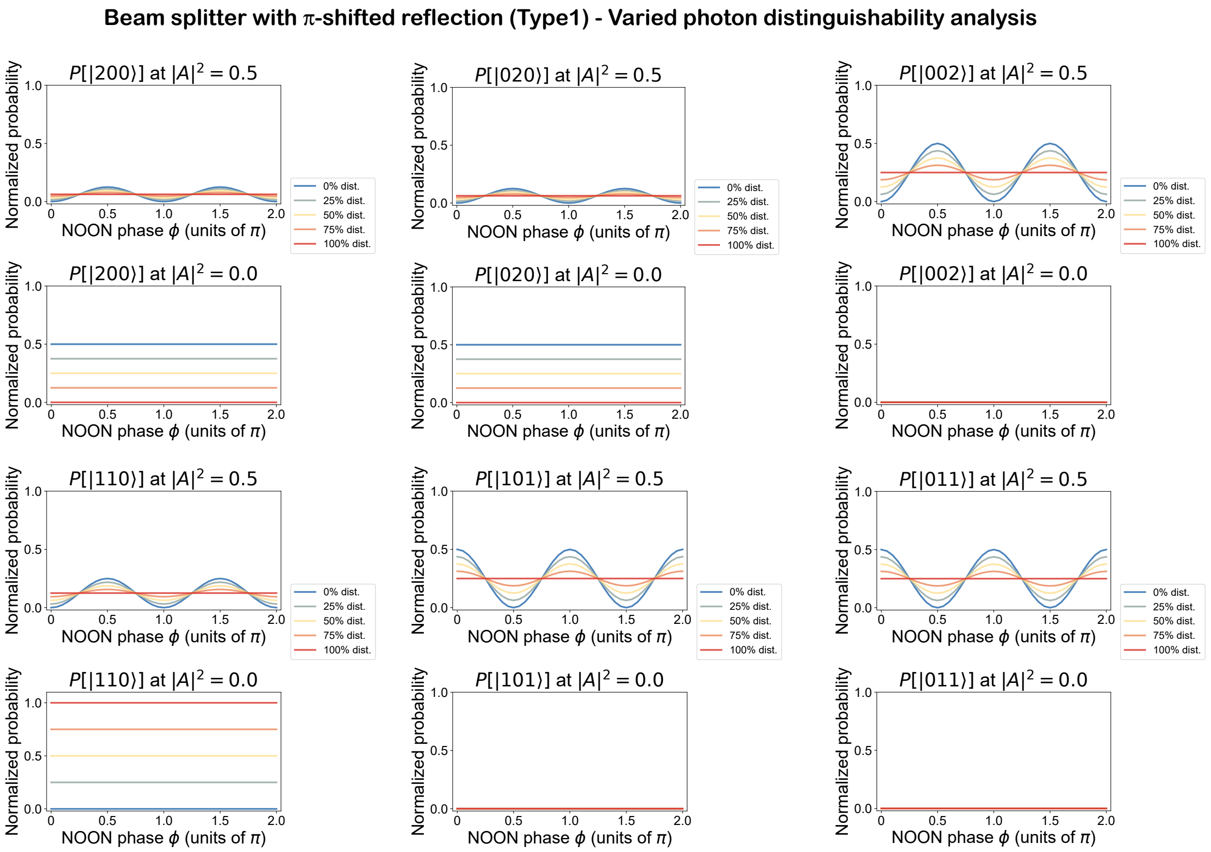

Supplementary Figure 11: **Theoretical analysis of photon distinguishability dependence of two-photon output probabilities for a Type 1 beamsplitter.** Normalized two-photon output probabilities as a function of the NOON phase ϕ (in units of π), shown for distinguishability values $d = 0, 0.25, 0.5, 0.75,$ and 1.0. Results are shown for a strongly absorbing device ($|A|^2 = 0.5$) and for a lossless device ($|A|^2 = 0$). Increasing distinguishability suppresses the phase-dependent modulation across all output channels, while incoherent background probabilities remain finite in the absorbing case.

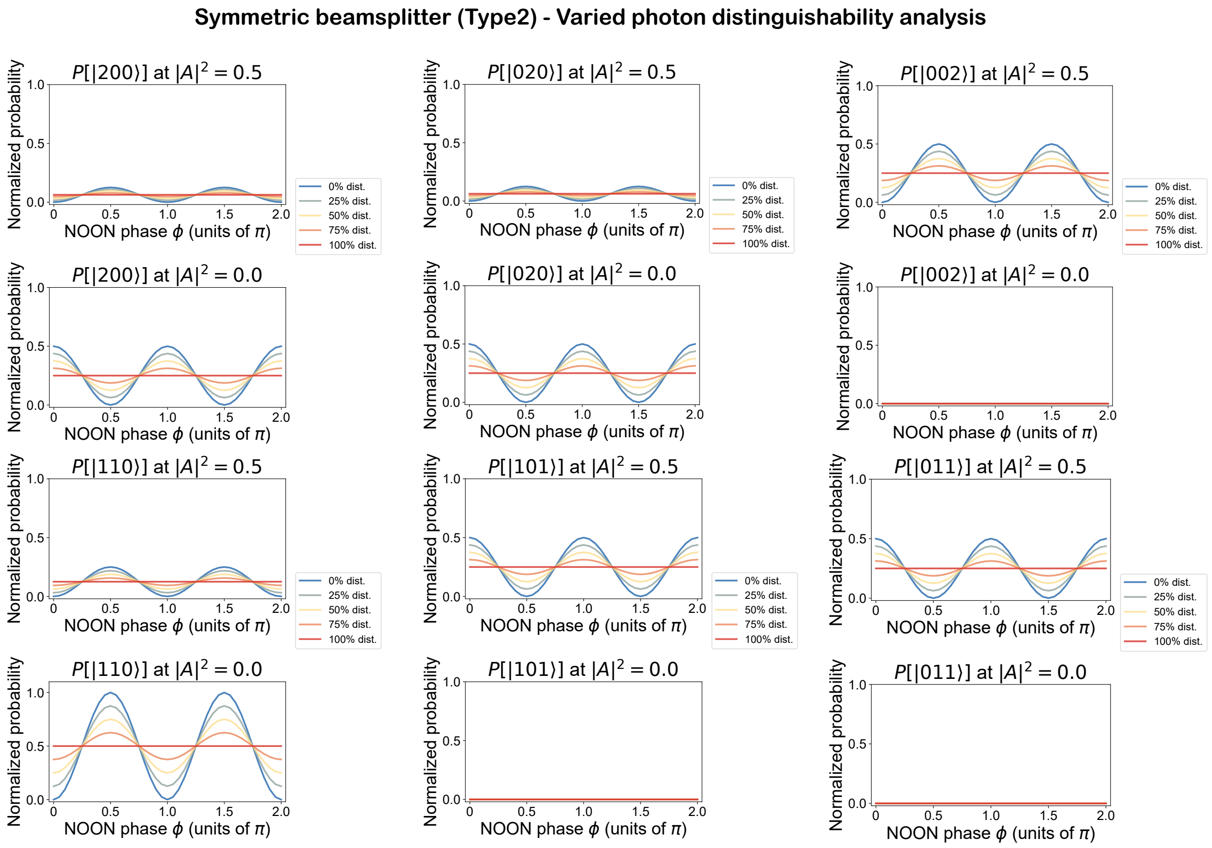

Supplementary Figure 12: **Theoretical analysis of photon distinguishability dependence of two-photon output probabilities for a Type 2 beamsplitter.** Same analysis as Supplementary Figure 11, but for the symmetric (Type 2) beamsplitter implementation. The interference fringes are continuously suppressed with increasing distinguishability and vanish in the fully distinguishable limit.

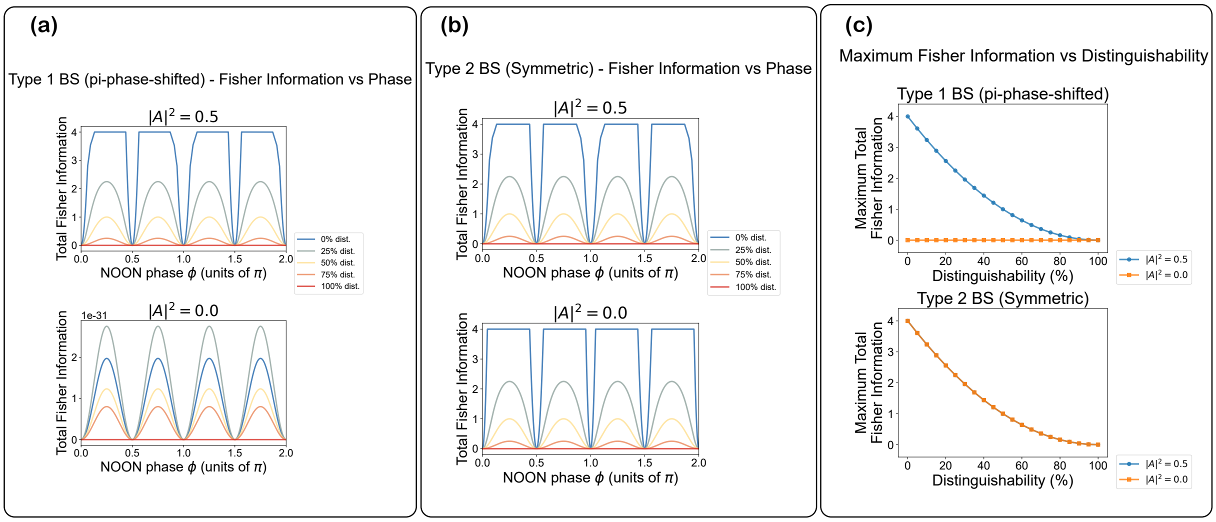

Supplementary Figure 13: **Theoretical analysis of the degradation of Fisher information under partial photon distinguishability.** (a) Total Fisher information $F_{tot}(\phi)$ versus NOON phase ϕ for the Type 1 beamsplitter. (b) Same as (a), but for the Type 2 beamsplitter. (c) Maximum Fisher information, optimized over ϕ, as a function of photon distinguishability d for both beamsplitter types and for $|A|^2 = 0.5$ and $|A|^2 = 0$. Increasing photon distinguishability leads to the reduction of phase sensitivity, vanishing in the fully distinguishable limit.

References

[1] Alexiev, C., Mak, J. C. C., Sacher, W. D. & Poon, J. K. S. Calibrating rectangular interferometer meshes with external photodetectors. *OSA Continuum* **4**, 2892–2904 (2021).

[2] Lin, S. *et al.* Power-efficient programmable integrated multiport photonic interferometer in cmos-compatible silicon nitride. *Photonics Research* **12**, A11–A20 (2024).

[3] Vetlugin, A. N. Coherent perfect absorption of quantum light. *Physical Review A* **104**, 013716 (2021).
